# Single-Cell RNA Sequencing Combined with MEBOCOST Reveals Alveolar Macrophage-Mediated Immunometabolic Communication Mechanisms in Sepsis-Induced Acute Lung Injury

**DOI:** 10.3390/genes17091019

**Published:** 2026-08-27

**Authors:** Lixia Zhao, Xin Liu, Yubang Hu, Yuming Yang, Yue Peng, Youtan Liu

**Affiliations:** 1 Shenzhen School of Clinical Medicine, Southern Medical University, Shenzhen 518110, China; 2 Department of Anesthesiology, Shenzhen Hospital, Southern Medical University, Shenzhen 518110, China; 3 Department of Anesthesiology, The Eighth Affiliated Hospital, Sun Yat-sen University, Shenzhen 518000, China

**Keywords:** sepsis-induced acute lung injury, Slc7a11, cystine metabolism, MEBOCOST, metabolite–sensor communication, alveolar macrophage, single-cell RNA sequencing

## Abstract

**Background:** Sepsis-induced acute lung injury (SI-ALI) leads to high mortality in critically ill patients, and no specific targeted treatments are available. Current studies mainly characterize intercellular crosstalk via protein–ligand–receptor frameworks, while metabolite-derived immune signal transmission is largely unclarified. Alveolar macrophages are located at the alveolar barrier and may act as central metabolic coordinators to trigger neutrophil-mediated lung inflammation, yet the complete metabolite signaling network has not been systematically mapped. **Methods:** We integrated single-cell and bulk transcriptomic datasets from CLP-induced septic mouse lungs. CellChat and MEBOCOST were jointly used to reconstruct protein–metabolite dual communication networks. LASSO regression and random forest were combined to screen core metabolic hub genes, followed by single-cell expression mapping and in vivo histology & qPCR validation. **Results:** We identified a pivotal alveolar macrophage–neutrophil immunometabolic axis dominated by iron-Slc40a1 and LTB4-Ltb4r1 signaling. Three hub genes (Pmvk, Slc2a1, Slc7a11) coordinately regulate inflammatory pathways, among which myeloid-enriched Slc7a11 balances cellular redox and paracrine inflammatory responses. **Conclusions:** This study establishes the first dual-layer cell communication atlas for SI-ALI, proposes a macrophage-centered metabolic inflammatory regulatory model, and highlights Slc7a11 as a potential therapeutic target for septic lung injury.

## 1. Introduction

Sepsis, defined as life-threatening organ dysfunction resulting from a dysregulated host response to infection, remains a global health emergency [1]. According to the Global Burden of Disease Study 2021, an estimated 166 million incident sepsis cases and 21.4 million sepsis-related deaths occurred worldwide in 2021 [2]. The lung is the most frequently injured organ in sepsis, and sepsis-induced acute lung injury (SI-ALI)—a major precipitant of acute respiratory distress syndrome (ARDS)—carries disproportionately high mortality [3,4]. The pathogenesis of SI-ALI involves an incompletely characterized interplay among alveolar epithelial injury, endothelial barrier disruption, and massive infiltration of neutrophils and monocyte-derived macrophages, which release pro-inflammatory cytokines, reactive oxygen species (ROS), and proteolytic enzymes [5,6]. Despite decades of investigation, no targeted pharmacotherapy has been approved for SI-ALI/ARDS, underscoring the urgent need to decipher the molecular circuitry that drives this lethal inflammatory cascade.

Emerging evidence positions metabolic reprogramming at the center of SI-ALI pathogenesis—not as a passive consequence of cellular activation, but as an active driver of immune effector function [7,8]. Pro-inflammatory macrophages undergo a GLUT1 (encoded by Slc2a1)-mediated glycolytic switch, diverting pyruvate from oxidative phosphorylation toward lactate production and fueling NLRP3 inflammasome activation and cytokine secretion [9,10]. Concurrently, ferroptosis—an iron-dependent, lipid-peroxidation-driven form of regulated cell death—has been implicated in alveolar epithelial destruction during SI-ALI, with the cystine/glutamate antiporter subunit SLC7A11 serving as a critical gatekeeper of glutathione synthesis and cellular redox homeostasis [11,12]. The mevalonate pathway, in which phosphomevalonate kinase (PMVK) catalyzes a key intermediate step, supplies isoprenoid precursors required for protein prenylation and has been linked to IL-1β hypersecretion in hyperinflammatory conditions [13,14]. Despite extensive characterization of each of these metabolic nodes in isolation, whether and how they coordinately contribute to the immune-metabolic dysregulation of SI-ALI remains unexplored.

The advent of single-cell RNA sequencing (scRNA-seq) has transformed our understanding of cellular heterogeneity in complex tissues, and its application to the injured lung has begun to resolve the cellular composition and transcriptional programs that underpin ALI/ARDS [15,16]. Recent large-scale lung cell atlases have catalogued numerous transcriptionally distinct epithelial, endothelial, stromal, and immune populations, revealing previously unrecognized transitional cell states and disease-specific activation signatures [17,18]. In the context of SI-ALI, scRNA-seq studies have delineated monocyte-to-macrophage differentiation trajectories, identified interferon-responsive and pro-fibrotic macrophage subsets, and characterized neutrophil heterogeneity during the acute inflammatory phase [19,20]. However, these analyses have predominantly relied on protein–ligand–receptor frameworks—such as CellChat and NicheNet—to infer intercellular communication, leaving the metabolic dimension, particularly how metabolites function as intercellular messengers between immune cells, largely unexplored.

Computational tools capable of inferring metabolite-mediated cell–cell communication from scRNA-seq data have recently been developed to address this gap. Among them, MEBOCOST integrates enzyme and transporter expression data with curated metabolite–sensor databases to quantitatively reconstruct metabolite-mediated communication networks [21], capturing signaling events—such as lipid, amino acid, and nucleotide metabolite exchanges—that are invisible to conventional protein–ligand–receptor pipelines. Importantly, combining MEBOCOST with protein-based communication inference (e.g., CellChat) provides a complementary, dual-layer portrait of intercellular signaling that neither approach can achieve alone. Such integration is particularly critical for the SI-ALI lung, where alveolar macrophages (AMs)—positioned at the air–tissue interface and endowed with extensive metabolic machinery—may serve as metabolic signal integrators that receive upstream cues and, in turn, dispatch metabolite signals that amplify neutrophil-driven inflammation. This metabolite-mediated AM–neutrophil communication axis, however, has not been systematically characterized.

The present study aims to integrate scRNA-seq profiling, CellChat-based protein communication inference, MEBOCOST-based metabolite communication reconstruction, and bulk transcriptome validation to systematically dissect the metabolite-mediated AM–neutrophil communication network in SI-ALI. By coupling dual machine learning feature selection (Random Forest and LASSO) with independent cohort validation, we further seek to identify key metabolic hub genes that govern this crosstalk. We hypothesize that AMs function as metabolic signal integrators whose reprogrammed metabolic output—channeled through specific transporters and enzymes—drives neutrophil inflammatory amplification, and that these metabolic hubs may represent actionable therapeutic targets for SI-ALI.

## 2. Materials and Methods

### 2.1. Data Acquisition

Raw single-cell RNA sequencing (scRNA-seq) data were retrieved from the Gene Expression Omnibus (GEO) repository hosted by the National Center for Biotechnology Information (NCBI; https://www.ncbi.nlm.nih.gov/geo/ (accessed on 25 March 2026)). The publicly available dataset GSE207651, which comprises single-cell transcriptomic profiles from murine (Mus musculus) lung tissues, was downloaded for bioinformatic analysis. Samples were grouped into sham-operated controls and CLP-induced sepsis groups; samples from different post-surgical time points were combined into the sepsis group because this study focused on disease-versus-control comparison rather than temporal dynamics.

For independent cohort validation, bulk RNA-seq data were obtained from GSE269740, comprising lung tissue transcriptomic profiles from three sham-operated controls and three CLP-induced sepsis mice. This dataset was used for immune infiltration deconvolution, GSEA, GSVA, and differential expression validation of the metabolic signature genes.

### 2.2. Single-Cell Analysis

Raw scRNA-seq data were processed using Seurat (v5.0.0) in R (v4.3.3). Quality control metrics—including nFeature_RNA, nCount_RNA, percent.mt, and percent.ribo—were assessed using violin plots and scatter plots; percent.ribo was inspected for quality monitoring but not used as a filtering criterion. Doublets were removed using scDblFinder (v1.16.0). Cells with nFeature_RNA < 200 or >4000, nCount_RNA > 25,000, or percent.mt > 20% were excluded, yielding 28,012 high-quality cells for downstream analysis.

The top 2000 highly variable genes (HVGs) were identified using the “vst” method, followed by log-normalization (LogNormalize, scale factor = 10,000) and scaling. The number of principal components (PCs) was determined using an elbow plot, and the first 20 PCs were used for Harmony (v1.2.0)-based batch correction. A shared nearest-neighbor (SNN) graph was constructed on the Harmony-adjusted embeddings, and cell clusters were identified using the FindClusters function at a resolution of 0.2. Two-dimensional visualization was performed using uniform manifold approximation and projection (UMAP).

Cluster-specific marker genes were identified using the Wilcoxon rank-sum test (FindAllMarkers, log_2_FC > 0.25, adjusted * *p* < 0.05). Each cluster was annotated as one of 12 major cell types based on canonical lineage markers. Marker gene expression was visualized using a dot plot, where dot size represents the proportion of expressing cells and color intensity corresponds to the mean expression level. Cell-type proportions across control and sepsis groups were calculated and displayed using stacked bar charts.

Gene Ontology (GO) biological process enrichment analysis was performed on cluster-specific marker gene sets using clusterProfiler (v4.10.1, adjusted * *p* < 0.05). Enrichment results were integrated with cell-type expression signatures and visualized as a heatmap.

### 2.3. Ligand–Receptor Interaction Analysis (CellChat)

Cell–cell communication networks were inferred using CellChat (v1.6.0) based on the Seurat-processed normalized count matrix and cell-type annotations generated above. Ligand–receptor interactions were evaluated using the curated mouse-specific CellChatDB reference database. Communication probabilities across all cell-type pairs were calculated via permutation test, and only statistically significant interactions (permutation test, * *p* < 0.05) were retained. The number and strength of intercellular interactions were summarized and visualized using circular network plots. Aggregate interaction strength was quantified to rank cell populations by their overall communication activity.

Outgoing and incoming signaling patterns were then extracted from CellChat outputs, and the relative contribution of individual pathways to total communication probability was calculated for each cell type and visualized using dot plots (dot size represents relative contribution score). Aggregated outgoing and incoming interaction strengths were displayed in a two-dimensional scatter plot to classify cell populations as dominant senders, receivers, or mediators.

The CXCL chemokine signaling network was further extracted and visualized using chord diagrams to illustrate the directionality and intensity of CXCL-mediated intercellular communication in sepsis-induced acute lung injury (SI-ALI). Differential expression of core CXCL family genes (Cxcl1–6, Cxcl10, Cxcl12, Cxcl16, Ackr3, and Pf4) was compared between control and sepsis groups and displayed using violin plots. Individual ligand–receptor pair contributions within the CXCL axis were quantified across all cell-type pairs and visualized using dot plots (dot size represents communication probability; color intensity represents statistical significance).

### 2.4. Metabolic Cell–Cell Communication Analysis

To complement ligand–receptor-based communication inference, metabolic cell–cell communication (mCCC) was analyzed using MEBOCOST (v1.0) based on the annotated scRNA-seq data. Metabolic activity scores were computed for individual cells from the expression profiles of metabolism-related genes using the default KEGG-derived metabolic pathway gene set implemented in MEBOCOST. Communication probabilities for metabolite–sensor pairs were calculated using the default MEBOCOST framework, and significant mCCC events were retained for downstream network analysis.

The number of mCCC events in which each cell type served as a metabolic sender or receiver was quantified and displayed as a paired bar chart to compare metabolic output versus uptake capacity across populations.

An aggregated mCCC network was then constructed, with nodes representing cell types and edges denoting significant metabolic communication events (edge thickness encodes event count; color intensity represents overall interaction score), and visualized using a circular diagram to reveal metabolite-driven crosstalk among immune and stromal compartments.

To resolve this landscape at finer resolution, a sender–receiver communication matrix was constructed, in which each entry represents the aggregated interaction score of a given cell-type pair, and visualized using a dot heatmap (dot size: event count; color: interaction score).

For pairs exhibiting prominent metabolic crosstalk, individual metabolite–sensor pairs were extracted, ranked by communication probability, and presented as a lollipop-style dot plot. Finally, the complete metabolic relay chain—spanning sender cell, secreted metabolite, sensor on receiver cells, and target receiver population—was reconstructed as a Sankey network diagram (edge width: communication score; node size: connectivity degree), enabling systematic tracing of metabolite flow from production to sensing within the intercellular ecosystem.

### 2.5. Machine Learning-Based Metabolic Gene Selection

To identify metabolic genes most predictive of SI-ALI status, a machine learning approach combining random forest (RF) and least absolute shrinkage and selection operator (LASSO) logistic regression was employed for feature selection, not for constructing a diagnostic classifier. Candidate metabolic genes were defined as those encoding enzymes and transporters implicated in the metabolite–sensor pairs identified by MEBOCOST metabolic crosstalk analysis. For model training, scRNA-seq expression data were aggregated to pseudobulk per sample by averaging normalized counts across all cells within each sample. Because the discovery cohort contained only three samples (one control and two sepsis), the models were used to rank and prioritize candidate genes rather than to derive a validated predictive signature.

Random forest classification was performed using the randomForest package (v4.7-1.1) in R, with disease status as the response variable and pseudobulk expression levels of candidate metabolic genes as predictors. Variable importance was assessed using mean decrease in Gini impurity (MeanDecreaseGini), and the top-ranked genes (ntree = 500, mtry = default) were retained. LASSO logistic regression was then applied using the glmnet package (v4.1-8) to further refine gene selection and avoid overfitting. Ten-fold cross-validation was used to determine the optimal regularization parameter λ at which the misclassification error was minimized (lambda.min). Genes with non-zero coefficients at the optimal λ were retained, and the regularization paths of all candidate genes across the full range of λ values were visualized to illustrate the stability of their contributions.

The top-ranked metabolic genes identified by random forest were intersected with those retained by LASSO at the optimal λ, and the overlapping genes were defined as the final candidate metabolic regulators for SI-ALI and visualized using a Venn diagram. Differential expression of each signature gene between control and sepsis groups was assessed using the GSE269740 validation cohort and a two-sided Wilcoxon rank-sum test, with statistical significance denoted as * *p* < 0.05 and ** *p* < 0.01, and displayed using grouped box plots. External validation served to confirm the directionality of expression changes identified in the discovery cohort, rather than to validate a trained classifier.

### 2.6. Immune Infiltration Analysis and Hub Gene–Immune Correlation Profiling

To characterize the SI-ALI immune microenvironment at the bulk-tissue level, immune cell infiltration was estimated using CIBERSORT (v1.03) deconvolution with the LM22 signature matrix applied to the GSE269740 bulk RNA-seq expression matrix. Relative proportions of 22 immune cell subpopulations were computed for each sample and displayed using stacked bar charts to compare compositional differences between control and sepsis groups. Pairwise Pearson’s correlation coefficients across all immune cell types were calculated and visualized using a heatmap to reveal co-infiltration patterns. The relative abundance of each subpopulation was then compared between control and sepsis groups using a two-sided Wilcoxon rank-sum test, with results displayed using grouped box plots (* *p* < 0.05; ** *p* < 0.01; ns, not significant). To further examine the association between the metabolic candidate metabolic regulators and the immune microenvironment, Pearson’s correlation coefficients were calculated between the expression levels of Pmvk, Slc2a1, and Slc7a11 and the infiltration scores of each immune cell type across all samples in the GSE269740 cohort. Correlation strength and direction were visualized using a heatmap annotated by statistical significance (* *p* < 0.05; ** *p* < 0.01).

### 2.7. Hub Gene–Immune Modulator Correlation Analysis

To evaluate the association between the metabolic hub gene signature (Pmvk, Slc2a1, Slc7a11) and immune regulatory networks, Pearson’s correlation coefficients were calculated between the expression levels of each signature gene (GSE269740 bulk expression matrix) and five curated immunological gene sets across all samples: chemokines, receptors, major histocompatibility complex (MHC) molecules, immunostimulators, and immunoinhibitors. These gene sets were obtained from the TISIDB database (http://cisitdb.cancer-pku.cn/ (accessed on 27 May 2026)). Correlation strength and statistical significance were visualized using dot plots, where dot color indicates correlation direction (red, positive; blue, negative), dot size reflects the absolute correlation coefficient, and asterisks indicate statistical significance (* *p* < 0.05; ** *p* < 0.01).

### 2.8. GSEA

Gene Set Enrichment Analysis (GSEA) was performed to characterize the biological pathways associated with each metabolic hub gene. Using the GSE269740 bulk expression matrix, all detected genes were ranked by their Pearson’s correlation coefficient with Pmvk, Slc2a1, and Slc7a11, respectively, and enrichment was assessed against the KEGG pathway database using the clusterProfiler package (v4.10.1) with 1000 phenotype permutations. Pathways meeting |normalized enrichment score| ≥ 1.0 and false discovery rate (FDR) q < 0.25 were considered significantly enriched; the dual thresholding criterion was applied to balance sensitivity for exploratory pathway discovery with specificity against spurious enrichment. The top three enriched pathways per hub gene were visualized using enrichment plots.

### 2.9. GSVA

Gene set variation analysis (GSVA) was performed to compare pathway activity with the expression of each metabolic hub gene. Using the GSE269740 bulk expression matrix, Pearson’s correlation coefficients were calculated between the expression level of each hub gene (Pmvk, Slc2a1, and Slc7a11) and GSVA enrichment scores for Hallmark gene sets (MSigDB) calculated using the GSVA package (v1.50.0). In addition, samples were divided into high- and low-expression groups according to median hub gene expression, and differential GSVA scores between these groups were assessed using linear models via limma (v3.58.1), with statistical significance defined as adjusted *p* < 0.05 after Benjamini–Hochberg correction. The resulting correlation coefficients and t-values were plotted to illustrate the magnitude and directionality of pathway perturbation.

### 2.10. Prediction of Upstream Regulatory Network

To investigate the upstream transcriptional and post-transcriptional regulatory mechanisms of the three metabolic signature genes (Pmvk, Slc2a1, Slc7a11), two bipartite regulatory networks were constructed, respectively.

For the transcription factor (TF) regulatory network, literature-curated TF–target interaction pairs of mouse genes were extracted from the TRRUST v2 database. TFs with experimentally documented regulatory relationships with Pmvk, Slc2a1, or Slc7a11 were retained for network construction, with TFs defined as source nodes and the three metabolic genes as target nodes.

For the microRNA (miRNA)–target regulatory network, high-confidence miRNA–target prediction data for mouse genes were retrieved from the miRWalk database. Candidate miRNAs predicted to target at least one of the three metabolic signature genes were included to build the interaction network, with miRNAs as source nodes and metabolic genes as target nodes.

Both networks were visualized using Cytoscape software (v3.10).

### 2.11. Single-Cell Metabolic Profiling and Gene–Pathway Association Analysis

To characterize single-cell metabolic activity, MEBOCOST—which estimates per-cell pathway scores from the expression of metabolic enzymes and transporters—was applied to the 28,012 high-quality cells retained after upstream preprocessing. For each metabolic hub gene (Pmvk, Slc2a1, Slc7a11), its expression was correlated using Pearson’s correlation with MEBOCOST pathway scores across all cells. Significant associations (FDR q < 0.05) were visualized using bubble plots, where color encodes the Pearson’s correlation coefficient and size reflects FDR-adjusted significance, with pathways grouped into five functional categories: inflammation, immune response, metabolism, signaling, and proliferation.

The expression distribution of each hub gene was visualized on the Harmony-corrected UMAP embedding, and cell-type-specific expression patterns were assessed across all 12 annotated cell populations using dot plots (percent expressed × average expression) and violin-jitter plots. Finally, differential expression between SI-ALI and sham-operated controls was evaluated within each cell population using the Wilcoxon rank-sum test via Seurat’s FindMarkers function and visualized using condition-stratified dot plots.

### 2.12. Establishment of the Sepsis-Induced Lung Injury Model

All animal experiments were approved by the Institutional Animal Care and Use Committee of The Eighth Affiliated Hospital, Sun Yat-sen University and conducted in strict accordance with relevant guidelines. Male C57BL/6 mice (8–10 weeks, 22–25 g) were acclimatized for 7 days under standard housing conditions (12 h light/dark cycle). Mice were fasted for 12 h preoperatively with free access to drinking water.

Sepsis was induced via cecal ligation and puncture (CLP). Briefly, mice were anesthetized with pentobarbital sodium (Sigma-Aldrich, St. Louis, MO, USA; 50 mg/kg, i.p.). Under sterile conditions, a 1.2 cm midline abdominal incision was made to expose the cecum. Half of the cecal length was ligated with 3-0 silk suture (Jinhuan Medical, Shanghai, China) without occluding mesenteric vessels. The ligated cecum was punctured twice with a 22-gauge needle, and a small amount of fecal content was extruded. The cecum was returned to the peritoneal cavity, and the abdominal wall was closed in layers with 4-0 sutures (Jinhuan Medical, Shanghai, China). Immediately after surgery, 1 mL pre-warmed sterile normal saline was administered subcutaneously for fluid resuscitation. Sham-operated mice underwent identical laparotomy and cecal exposure without ligation or puncture. All mice were euthanized 24 h post-surgery for lung tissue collection.

### 2.13. Hematoxylin–Eosin (H&E) Staining

Lung tissues were fixed in 4% paraformaldehyde (Biosharp, Hefei, China) at room temperature for 24 h, dehydrated through gradient ethanol, and embedded in paraffin. Serial 4 μm sections were cut and stained with H&E. Pulmonary pathological changes were observed and photographed under a light microscope (Leica, Wetzlar, Germany).

### 2.14. RNA Extraction and Quantitative Real-Time PCR

Total RNA was extracted from lung tissues (*n* = 3 mice per group) using the FastPure Cell/Tissue Total RNA Isolation Kit V2 (RC112-01, Vazyme, Nanjing, China) according to the manufacturer’s instructions. RNA concentration and purity were assessed using a NanoDrop 2000 spectrophotometer (Thermo Fisher Scientific, Waltham, MA, USA). Genomic DNA elimination and complementary DNA (cDNA) synthesis were performed with 1 μg of total RNA using the HiScript III RT SuperMix for qPCR (+gDNA wiper) (R323-01, Vazyme, Nanjing, China) under standardized reaction conditions.

Quantitative real-time PCR (qRT-PCR) was carried out using the Hieff UNICON Advanced qPCR SYBR Master Mix (11151ES10, YEASEN, Shanghai, China) on the ABI 7500 Real-Time PCR System (Applied Biosystems, Thermo Fisher Scientific, Waltham, MA, USA). The amplification protocol consisted of initial denaturation at 95 °C for 30 s, followed by 40 cycles of 95 °C for 5 s and 60 °C for 30 s. Melting curve analysis was conducted at the end of amplification to verify PCR product specificity.

Transcriptional levels of the target genes and of TNF-α, IL-6, IL-1β, CXCL1, and CXCL2 were quantified via the 2^−ΔΔCt^ method with GAPDH as the endogenous reference. Primer sequences are detailed in Table A1 (Appendix A).

### 2.15. Virtual Knockout Simulation Using scTenifoldKnk

To investigate the functional impact of the identified hub genes, in silico knockout simulations were performed using scTenifoldKnk (v1.0.1) [22], which constructs gene regulatory networks from single-cell transcriptomic data and identifies differentially regulated genes following target gene deletion through manifold alignment and chi-square statistics. The single-cell expression matrix was subsampled via stratified sampling, and virtual knockout was performed separately for Pmvk, Slc2a1, and Slc7a11. Perturbed genes were converted to Entrez gene identifiers and subjected to Gene Ontology Biological Process (GO BP) enrichment analysis with Benjamini–Hochberg FDR correction (adjusted *p* < 0.05). Volcano plots were generated to visualize differentially regulated genes.

### 2.16. Statistical Analysis

All statistical analyses were performed in R (v4.3.3). For comparisons between two groups, the two-sided Student’s t-test was used unless otherwise specified. Pearson’s correlation was used for all linear association analyses. Multiple testing correction was applied using the Benjamini–Hochberg method to control the false discovery rate (FDR), with adjusted *p* < 0.05 considered statistically significant.

## 3. Results

### 3.1. Preliminary Data Processing

To construct a reliable single-cell RNA-seq (scRNA-seq) atlas of sepsis-induced acute lung injury (SI-ALI), we first performed standardized quality control (QC) on transcriptomic data derived from one sham-operated control lung sample and two SI-ALI lung samples. Distributions of nCount_RNA, nFeature_RNA, mitochondrial gene proportion (percent.mt) and ribosomal gene proportion (percent.ribo) were highly consistent across all three specimens, indicating good inter-sample reproducibility (Figure S1A). Correlation analysis showed a strong positive association between nFeature_RNA and nCount_RNA (R = 0.9), while percent.mt and percent.ribo barely correlated with sequencing depth (R = 0.02 and R = 0.01, respectively) (Figure S1B). We filtered low-quality cells and doublets identified via scDblFinder according to universal single-cell QC thresholds: nFeature_RNA < 200 or >4000, nCount_RNA > 25,000, percent.mt > 20%. After filtering, a total of 28,012 high-quality cells were retained for downstream analysis.

Following normalization and scaling, 2000 highly variable genes (HVGs) were identified. Top HVGs included canonical pulmonary epithelial markers (Sftpc, Scgb1a1, Sftpa1, Sftpb, Scgb3a2) and the inflammatory chemokine Cxcl13, reflecting the native cellular composition of lung tissue and the characteristic inflammatory transcriptional signature triggered by sepsis (Figure S1C). An elbow plot indicated that the first 20 principal components (PCs) captured the majority of biologically meaningful transcriptional variation within the dataset (Figure S1D). Pre-integration PCA revealed obvious sample-specific clustering indicative of technical batch effects, which were effectively eliminated after Harmony integration (Figure S1E,F). In summary, rigorous QC and batch correction removed technical noise, generating an integrated high-quality dataset that supported subsequent cell-type annotation and MEBOCOST-mediated metabolite-transcriptome joint signaling analysis.

### 3.2. Single-Cell Transcriptomic Landscape of SI-ALI

**Figure 1:**
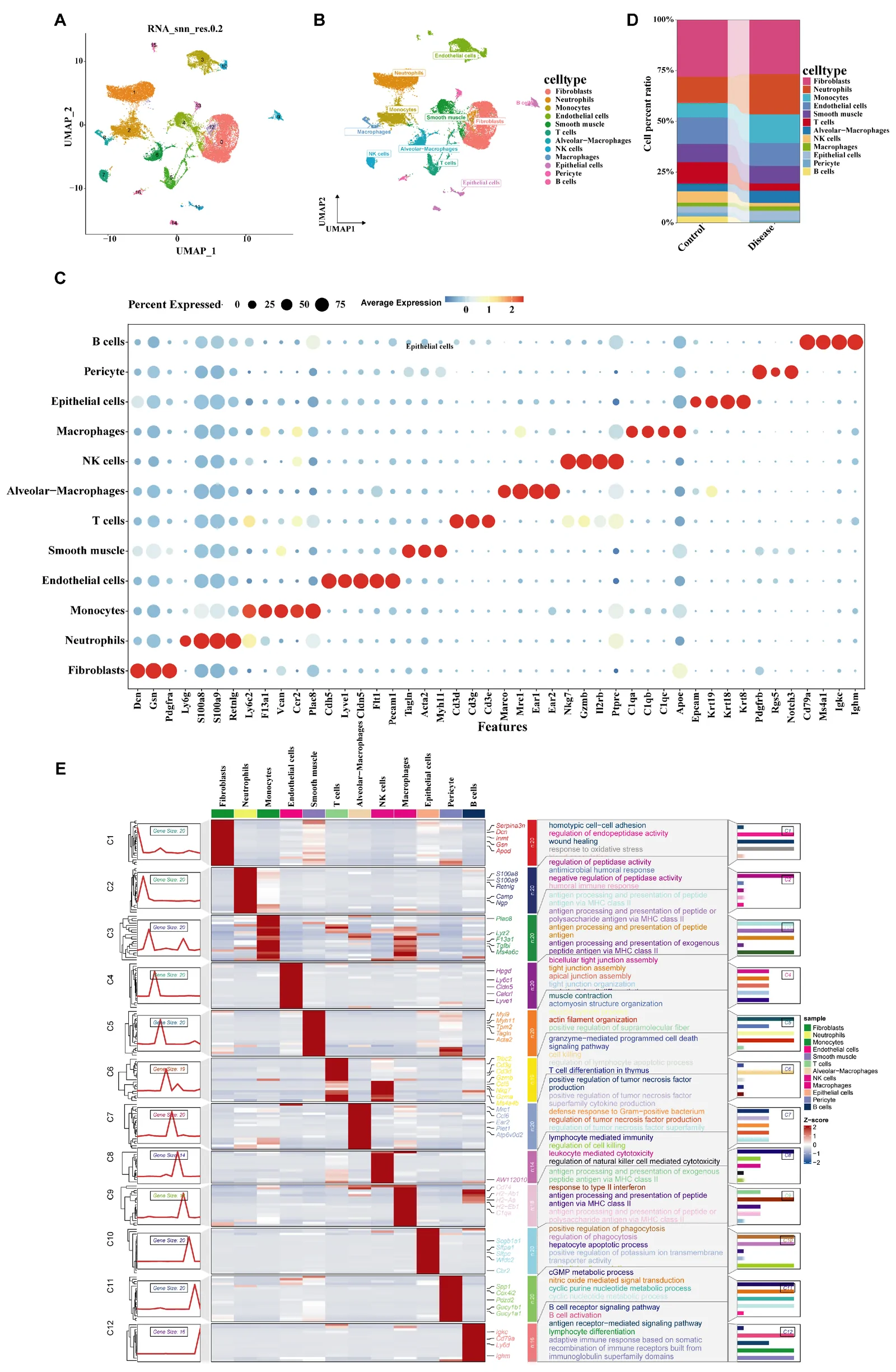
Single-cell transcriptomic atlas of the SI-ALI microenvironment. (**A**) UMAP visualization of 28,012 high-quality cells following dimensionality reduction and Harmony-based batch correction, revealing 17 transcriptionally distinct clusters. (**B**) Cell-type annotation of 12 major populations based on canonical lineage markers, projected onto the UMAP embedding. (**C**) Bubble plot showing the expression of cell-type-specific marker genes across all clusters. (**D**) Stacked bar chart comparing cell-type proportions between control and sepsis samples, illustrating substantial expansion of neutrophils and monocytes and depletion of adaptive lymphoid cells (T cells, NK cells, and B cells) in the sepsis group. (**E**) Heatmap of GO enrichment analysis based on cluster-specific marker genes, highlighting innate immune activation, antigen presentation.

We next applied the Seurat v5 pipeline to characterize the full cellular heterogeneity of lung tissue under SI-ALI. After QC, dimensionality reduction and Harmony batch correction, UMAP visualization resolved 17 transcriptionally distinct cell clusters (Figure 1A). Based on established lineage marker genes, these clusters were annotated into 12 major lung cell populations: neutrophils, monocytes, macrophages, alveolar macrophages (AMs), NK cells, T cells, B cells, fibroblasts, endothelial cells, smooth muscle cells, epithelial cells and pericytes (Figure 1B). The identity of each cell population was further validated by cell-type-specific marker gene expression across all clusters (Figure 1C).

Comparative analysis of cell-type proportions uncovered profound cellular remodeling induced by septic injury. Neutrophils and monocytes were markedly expanded in SI-ALI lungs, whereas adaptive immune lymphocytes (T, NK and B cells) were drastically depleted; structural stromal cells remained relatively stable (Figure 1D). This pattern recapitulates the hallmark pathology of clinical SI-ALI: overwhelming innate immune infiltration combined with adaptive immune paralysis. To dissect functional features of distinct cell subsets, we conducted GO enrichment analysis on cluster-specific marker genes. The enrichment heatmap demonstrated that innate immune populations (neutrophils, monocytes, alveolar macrophages) were highly enriched in pathways governing innate immune activation, antigen presentation and pro-inflammatory cytokine secretion, while stromal cell clusters were dominated by tissue remodeling and cell adhesion-related biological processes (Figure 1E).

### 3.3. Cell–Cell Communication Networks in SI-ALI

**Figure 2:**
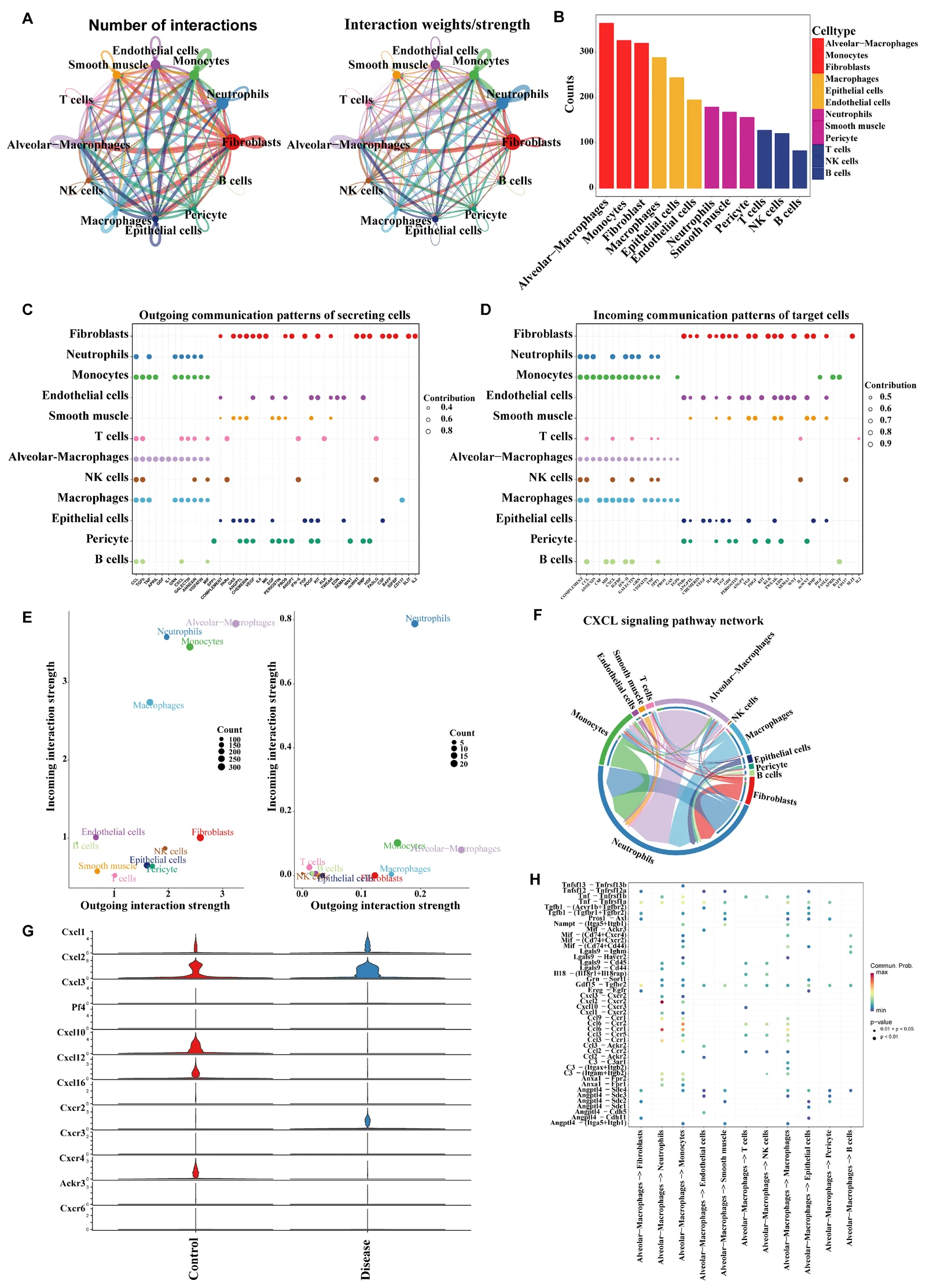
Cell–cell communication landscape and CXCL chemokine signaling in SI-ALI. (**A**) Circular network diagrams depicting ligand–receptor interactions among 12 cell populations. (**B**) Ranking of cell populations by overall interaction activity, with Alveolar macrophages, monocytes, macrophages, and fibroblasts showing the highest communicative activity. (**C**) Pathway-level heatmap of outgoing communication patterns, identifying fibroblasts, neutrophils, and alveolar macrophages as the dominant signal senders. (**D**) Pathway-level heatmap of incoming communication patterns, highlighting fibroblasts, neutrophils, and endothelial cells as the major signal receivers. (**E**) Two-dimensional scatter plot of aggregated outgoing versus incoming interaction strengths across all pathways (left) and restricted to inflammatory and chemokine pathways (right), illustrating functional divergence among cell populations. (**F**) Chord diagram illustrating CXCL pathway-mediated signaling, with neutrophils, monocytes, and macrophages as primary senders targeting fibroblasts, epithelial cells, and endothelial cells. (**G**) Violin plots showing upregulation of Cxcl1, Cxcl2, and Cxcr2 in the sepsis group, indicative of CXCL–Cxcr2 axis activation. (**H**) Ligand–receptor pair analysis confirming predominant Cxcl1–Cxcr2 and Cxcl2–Cxcr2 interactions from alveolar macrophages to neutrophils, underscoring a macrophage-driven chemotactic circuit in SI-ALI.

To decode intercellular signaling circuits driving SI-ALI pathogenesis, we constructed ligand–receptor communication networks using the CellChat algorithm. Circular network plots demonstrated extensive intercellular signaling interactions across all 12 annotated lung cell populations (Figure 2A). Quantification of total signal activity ranked alveolar macrophages, monocytes, macrophages and fibroblasts as the top four communicatively active cell types (Figure 2B).

Pathway-level decomposition of outgoing signals revealed fibroblasts, neutrophils and alveolar macrophages as the primary signal senders, which dominated secretion of inflammatory and chemokine ligands (Figure 2C). In contrast, fibroblasts, neutrophils and endothelial cells represented the major signal-receiving populations (Figure 2D). Two-dimensional projection integrating total incoming and outgoing signal strengths showed fibroblasts with prominent ligand secretion activity, while neutrophils and alveolar macrophages functioned as primary signal receivers and senders, respectively. When restricting analysis to inflammatory and chemokine pathways, overall interaction strength decreased globally, yet functional divergence persisted: neutrophils remained dominant signal receivers, and monocytes together with alveolar macrophages constituted the principal signal sources (Figure 2E).

The CXCL chemokine axis stood out as a core mediator of immune-stromal crosstalk. Chord diagrams illustrated widespread CXCL-mediated signaling originating from neutrophils, monocytes and macrophages and targeting fibroblasts, epithelial and endothelial cells (Figure 2F). Differential expression analysis confirmed significant upregulation of Cxcl1, Cxcl2 and their shared receptor Cxcr2 in SI-ALI samples relative to controls, demonstrating robust activation of the pro-inflammatory CXCL-Cxcr2 axis during septic lung injury (Figure 2G). Further ligand–receptor pair enrichment analysis verified that Cxcl1-Cxcr2 and Cxcl2-Cxcr2 interactions were preferentially enriched along the alveolar macrophage-to-neutrophil signaling direction, identifying a macrophage-originated chemotactic circuit responsible for massive neutrophil recruitment in SI-ALI progression (Figure 2H).

### 3.4. Metabolic Cell–Cell Communication Landscape in SI-ALI

**Figure 3:**
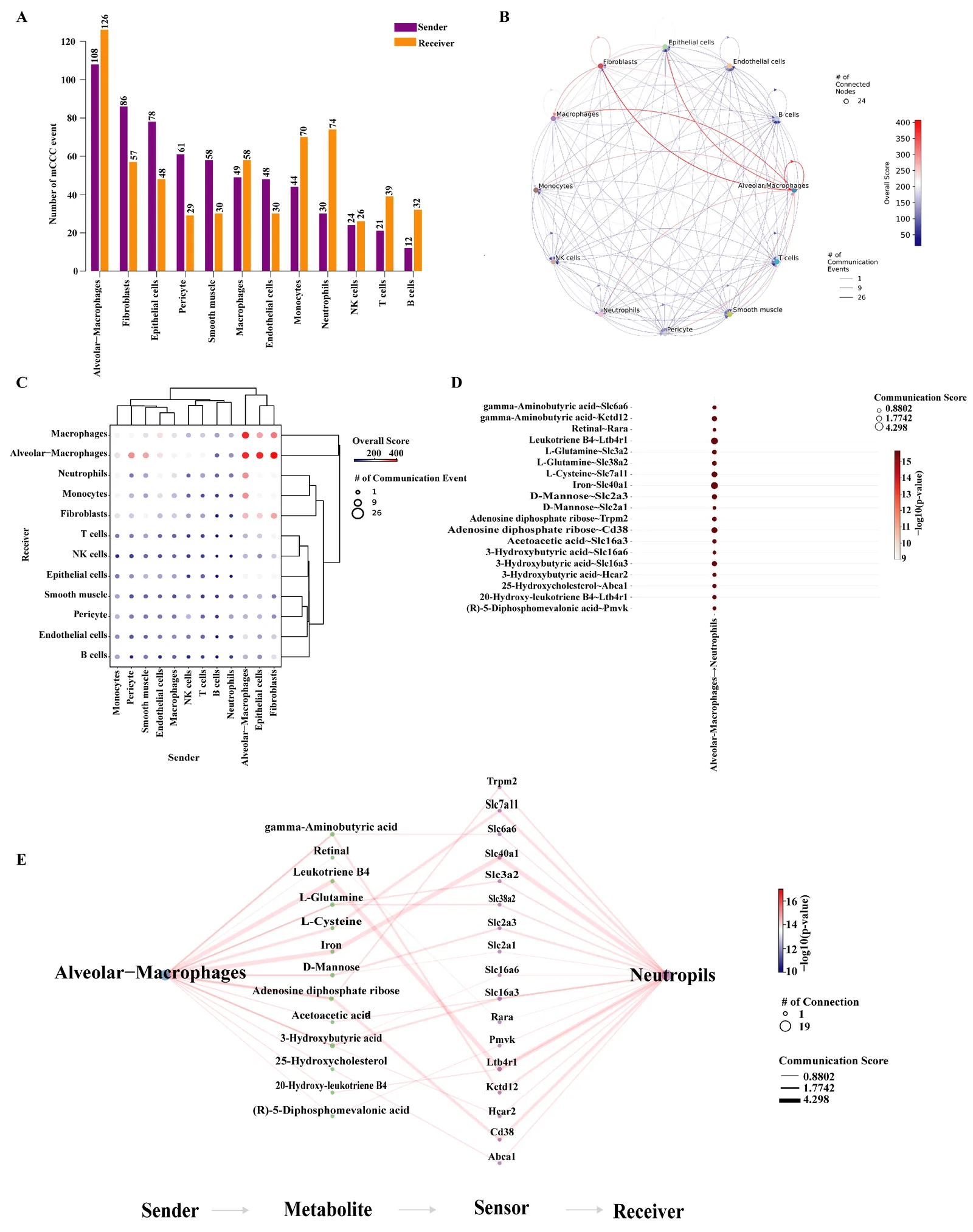
mCCC landscape and alveolar macrophage-centered signaling in SI-ALI. (**A**) Quantification of mCCC events identifying alveolar macrophages as dominant bidirectional signaling hubs, with fibroblasts and epithelial cells as primary metabolic senders and neutrophils and monocytes as major receivers. (**B**) Reconstructed interaction network positioning alveolar macrophages at the topological center. (**C**) Metabolic communication heatmap showing the highest outgoing and incoming interaction strengths in alveolar macrophages, with prominent autocrine activity and crosstalk with macrophages. (**D**) Ranking of the top 20 metabolite–receptor pairs in the alveolar macrophage-to-neutrophil axis, with Iron–Slc40a1 and leukotriene B4–Ltb4r1 as the most dominant pairs. (**E**) Bipartite signaling network illustrating the complete metabolite–receptor signal cascade from alveolar macrophages to neutrophils.

We implemented MEBOCOST to predict metabolite–receptor mediated cell–cell communication (mCCC) and resolve metabolic signal flow within the SI-ALI microenvironment. Global quantification of mCCC events identified alveolar macrophages as the central bidirectional metabolic signaling hub. Fibroblasts and epithelial cells acted as major metabolic substrate suppliers, while neutrophils and monocytes were the primary metabolic signal receivers (Figure 3A). Topological reconstruction of the metabolic interaction network further supported the central regulatory position of alveolar macrophages (Figure 3B).

Heatmap quantification of directional metabolic signal strength revealed that alveolar macrophages possessed the strongest incoming and outgoing metabolic signals, accompanied by robust autocrine signaling and extensive cross-talk with infiltrating macrophage subsets (Figure 3C). Given that neutrophils serve as primary effector cells mediating pulmonary inflammatory damage, we prioritized the top 20 metabolite–receptor pairs regulating AM–neutrophil communication. Iron-Slc40a1 and leukotriene B4 (LTB4)-Ltb4r1 represented the two strongest signaling axes, followed by GABA, L-glutamine, L-cysteine and 3-hydroxybutyrate-mediated metabolic communication (Figure 3D). Bipartite network visualization reconstructed the complete signaling cascade, illustrating that alveolar macrophages are predicted to secrete these metabolites, which may bind corresponding receptors on neutrophils and potentially propagate inflammatory metabolic signals (Figure 3E).

Notably, all metabolic communication outputs are computationally inferred solely from transcriptomic profiles without metabolic flux validation; these findings suggest but do not prove the proposed signaling axes. Collectively, these analyses suggest alveolar macrophages as a central integrator of metabolic signaling in septic lung tissue and identify iron-Slc40a1 and LTB4-Ltb4r1 as candidate immune-metabolic signaling axes warranting further experimental validation.

### 3.5. Machine Learning-Based Identification of Key Metabolic Hub Genes for SI-ALI

To screen functionally vital metabolic genes derived from mCCC prediction, we combined two complementary machine learning algorithms: random forest (RF) and least absolute shrinkage and selection operator (LASSO) regression. RF quantifies the predictive importance of all candidate genes by mean decrease in Gini impurity, whereas LASSO eliminates redundant variables to generate robust minimal signature sets. The intersection of genes prioritized by both algorithms was defined as core hub genes, using RF and LASSO for feature prioritization rather than predictive model development, to reduce single-model bias.

**Figure 4:**
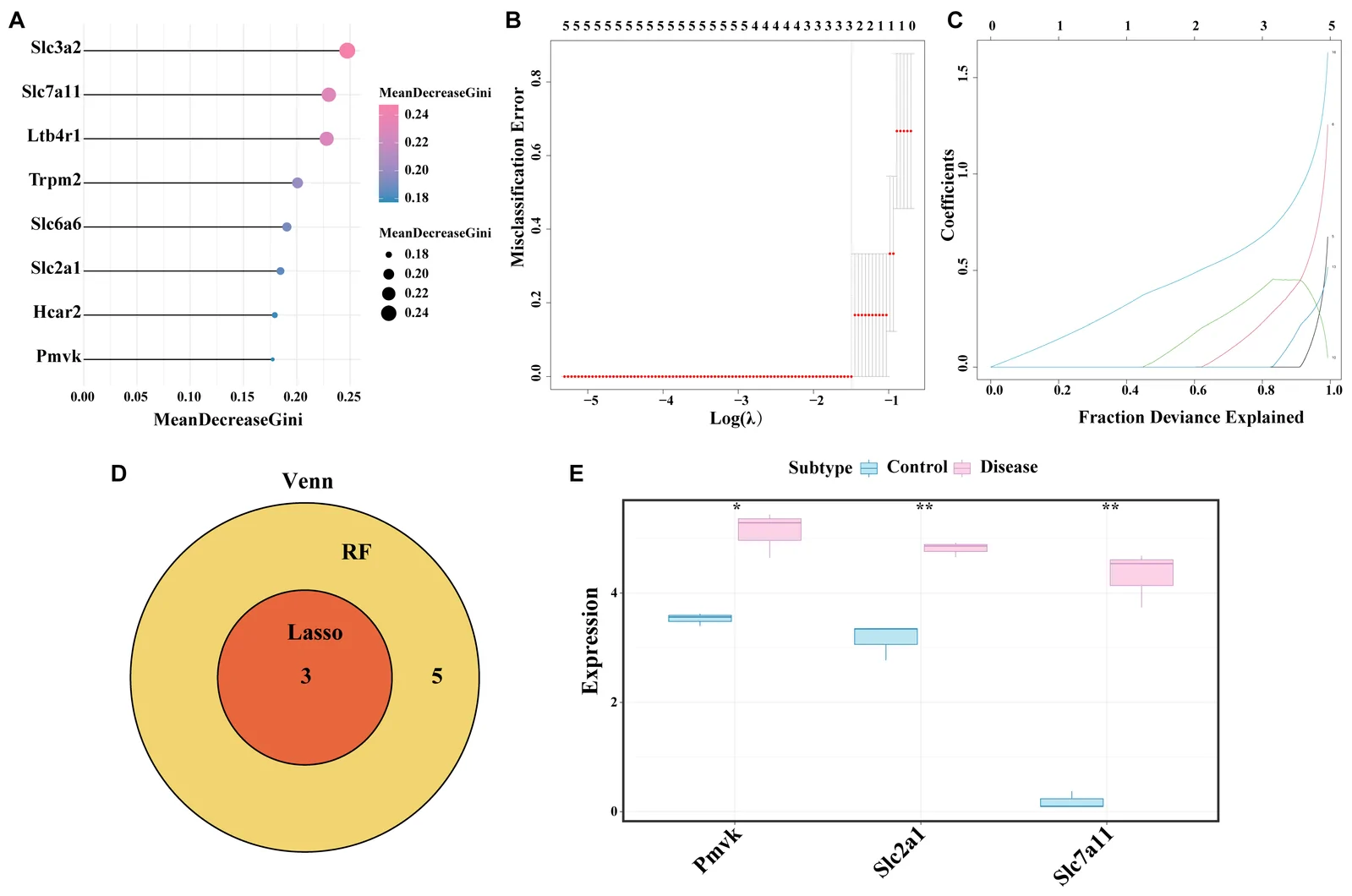
Identification of hub metabolic genes in SI-ALI via dual machine learning algorithms. (**A**) RF model ranking candidate genes by mean decrease in Gini impurity, identifying the top eight feature-important genes. (**B**) LASSO regression with 10-fold cross-validation selecting three genes with non-zero coefficients at optimal λ_min: Pmvk, Slc2a1, and Slc7a11. (**C**) LASSO coefficient trajectory plot, in which each colored line indicates the coefficient trajectory of one candidate gene as λ changes, confirming the largest absolute coefficient values for the three retained genes. (**D**) Venn diagram showing the intersection of RF- and LASSO-screened genes, yielding three consensus hub genes. (**E**) Differential expression of Pmvk, Slc2a1, and Slc7a11 between SI-ALI and healthy control samples in an independent cohort, demonstrating significant upregulation in the SI-ALI group.

The RF model output eight top-ranked candidate metabolic genes according to feature importance scores (Figure 4A). Parallel LASSO regression coupled with 10-fold cross-validation identified the optimal regularization parameter λ_min, filtering three genes with non-zero regression coefficients: Pmvk, Slc2a1, and Slc7a11 (Figure 4B). LASSO coefficient trajectory plots further verified that these three genes exhibited the largest absolute regression weights among all input variables (Figure 4C). Intersection analysis of RF and LASSO screening results yielded three overlapping hub genes (Figure 4D). Independent bulk transcriptomic cohort validation confirmed that Pmvk, Slc2a1 and Slc7a11 were all significantly upregulated in SI-ALI lung tissue compared with healthy controls (*p* < 0.01). Taken together, this dual-algorithm screening combined with external cohort validation identifies a triad of metabolic hub genes implicated in immune-metabolic cross-talk within inflamed pulmonary tissue during sepsis.

### 3.6. Immune Microenvironment Remodeling in SI-ALI and Its Association with Metabolic Hub Genes

**Figure 5:**
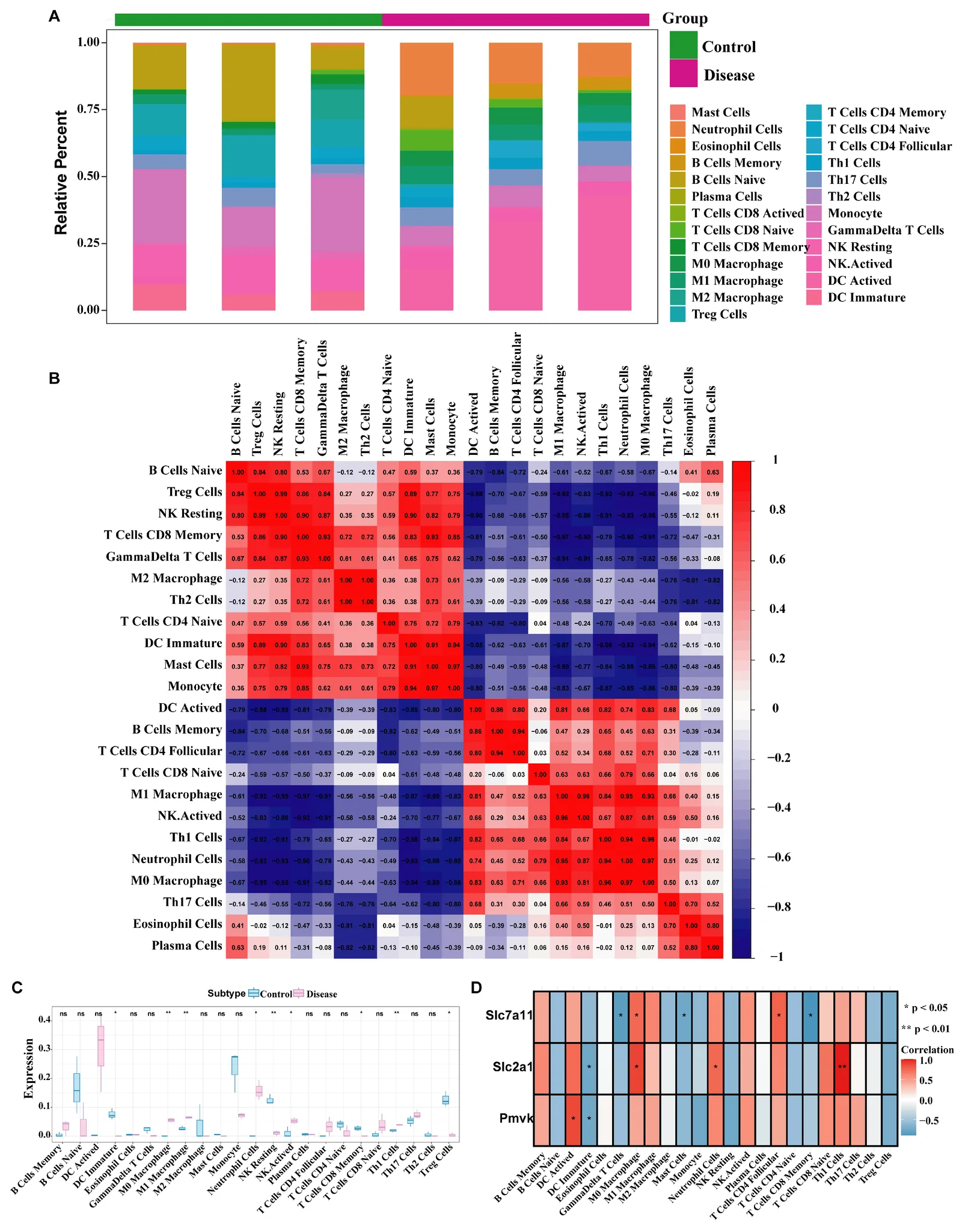
Bulk RNA-seq deconvolution of immune cell infiltration and correlation with hub gene expression in SI-ALI. (**A**) Stacked bar plot depicting immune cell composition estimated by CIBERSORT, showing marked enrichment of neutrophils, M0 macrophages, and Th17 cells alongside depletion of M2 macrophages, CD8^+^ memory T cells, and Tregs in SI-ALI samples. (**B**) Pearson’s correlation heatmap of 22 immune cell subsets, revealing a dichotomous pattern in which pro-inflammatory and anti-inflammatory populations form mutually inverse correlation modules. (**C**) Quantitative comparison of infiltration proportions across all 22 immune cell subtypes between control and SI-ALI groups, confirming significant enrichment of pro-inflammatory myeloid populations and reduction in resting lymphocyte subsets. (**D**) Correlation matrix between the three hub genes (Slc2a1, Slc7a11, Pmvk) and 22 immune cell subsets, highlighting distinct immune-regulatory associations for each hub gene.

We performed bulk RNA-seq-based immune infiltration analysis to validate single-cell immune remodeling observations at the tissue level. Stacked bar charts revealed striking compositional shifts between control and SI-ALI groups (Figure 5A). Neutrophils, which were rare in healthy lungs, accumulated massively in septic tissue alongside M0 macrophages and Th17 cells. By contrast, anti-inflammatory subsets including M2 macrophages, CD8^+^ memory T cells and T regulatory cells (Tregs) were markedly depleted in SI-ALI specimens.

Quantitative comparison across all 22 annotated immune cell subtypes further confirmed this pattern (Figure 5C). Pro-inflammatory myeloid populations (M0/M1 macrophages, neutrophils, Th17 cells) were significantly enriched in SI-ALI samples, whereas resting NK cells and naive CD4^+^ T cells showed reduced infiltration; other resting lymphocyte subsets displayed no statistically significant intergroup differences. Pearson’s correlation analysis uncovered a dichotomous immune correlation pattern: anti-inflammatory cell subsets formed an internally positively correlated module that broadly negatively correlated with pro-inflammatory populations, consistent with widespread immune microenvironment remodeling in septic lungs (Figure 5B). Neutrophils displayed strong positive correlations with all pro-inflammatory subtypes and consistent negative associations with anti-inflammatory immune cells.

We next evaluated linear correlations between the three metabolic hub genes and infiltration levels of all 22 immune cell subtypes (Figure 5D). Slc2a1 expression positively correlated with M0 macrophages, neutrophils and Th1 cells, while negatively associated with immature DCs, implying its involvement in pro-inflammatory myeloid recruitment. Slc7a11 was positively correlated with M0 macrophages and follicular CD4^+^ T cells, and negatively correlated with γδ T cells, mast cells and CD8^+^ memory T cells, suggesting its capacity to suppress cytotoxic and innate lymphocyte infiltration. Pmvk showed positive correlation with activated DCs and negative correlation with immature DCs, implying that Pmvk may participate in the regulation of DC maturation in inflamed lung tissue.

### 3.7. Metabolic Hub Genes Are Broadly Linked to Immune Regulatory Networks

**Figure 6:**
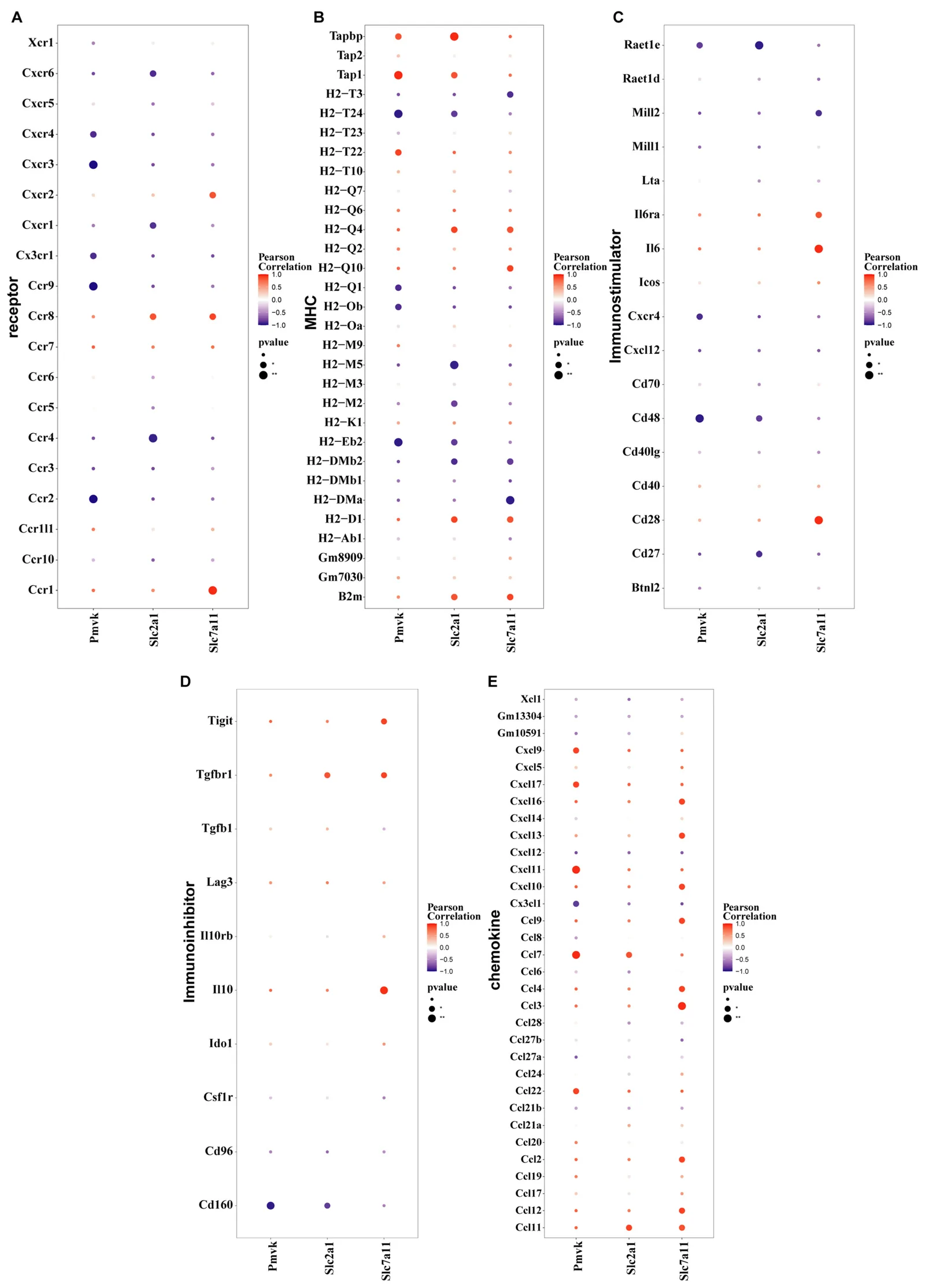
Correlation of hub metabolic genes with functional immune gene sets in SI-ALI. (**A**) Pearson’s correlation heatmap between Pmvk, Slc2a1, and Slc7a11 and chemokine receptor genes, with Slc7a11 showing the broadest positive associations. (**B**) Correlation profiles of the three hub genes with MHC-related genes, revealing divergent patterns across gene sets. (**C**) Correlation analysis with immunostimulatory molecules, highlighting Slc7a11 as the predominant positive regulator. (**D**) Correlation analysis with immunoinhibitory molecules, with Slc7a11 maintaining positive associations including Il10 and Tgfbr1. (**E**) Network visualization of hub gene connectivity across all five functional immune gene sets, with the chemokine panel exhibiting the widest connectivity.

Pearson’s correlation analysis was performed to assess associations between the three hub genes and five key immune functional gene panels (chemokine receptors, MHC molecules, immunostimulatory factors, immunoinhibitory factors, chemokines). In the chemokine receptor panel, Slc7a11 showed strong positive correlations with Ccr1 and moderate positive associations with Cxcr2 and Ccr8, alongside negative correlation with Cxcr6. Slc2a1 positively correlated with Ccr8, Ccr7 and negatively correlated with Cxcr6, Ccr4. Pmvk displayed prominent negative correlations with Ccr9, Ccr2, Cxcr4 and Cxcr3 (Figure 6A).

Major histocompatibility complex (MHC) gene correlations showed divergent patterns across the three genes (Figure 6B). Slc2a1 exhibited broad positive correlations with antigen-processing genes including Tapbp, H2-Q4, H2-D1, B2m, while it was negatively correlated with multiple inhibitory MHC loci such as H2-M5, H2-Eb2. Slc7a11 was positively associated with B2m, H2-Q4, H2-Q10 and negatively correlated with H2-DMa. Pmvk predominantly showed inverse correlations with MHC-related transcripts, particularly H2-T24 and H2-Eb2.

Within immunostimulatory gene sets, only Slc7a11 exhibited widespread positive correlations with Il6, Cd28 and Il6ra, alongside a moderate negative correlation with Mill2. Pmvk and Slc2a1 mostly displayed negative associations with immunostimulatory markers including Cd48 and Raet1e (Figure 6C). For immunoinhibitory genes, Slc7a11 maintained a strong positive correlation with Il10 and moderate positive associations with Tigit and Tgfbr1. Pmvk and Slc2a1 were negatively correlated with Cd160, and both shared a positive correlation with Tgfbr1 (Figure 6D). Among all immune panels, chemokine genes showed the broadest connectivity with the three metabolic hubs (Figure 6E). Slc7a11 displayed extensive positive correlations across CXC and CC chemokine families. Pmvk had sparse correlations, featuring a negative association with Cx3cl1 and positive correlations with Cxcl11, Ccl7, Cxcl9, Ccl22. Slc2a1 positively correlated with Ccl7 and Ccl11, and was moderately associated with Cxcl9. All three hub genes shared positive correlations with Ccl7 and Cxcl9.

### 3.8. GSEA and GSVA

**Figure 7:**
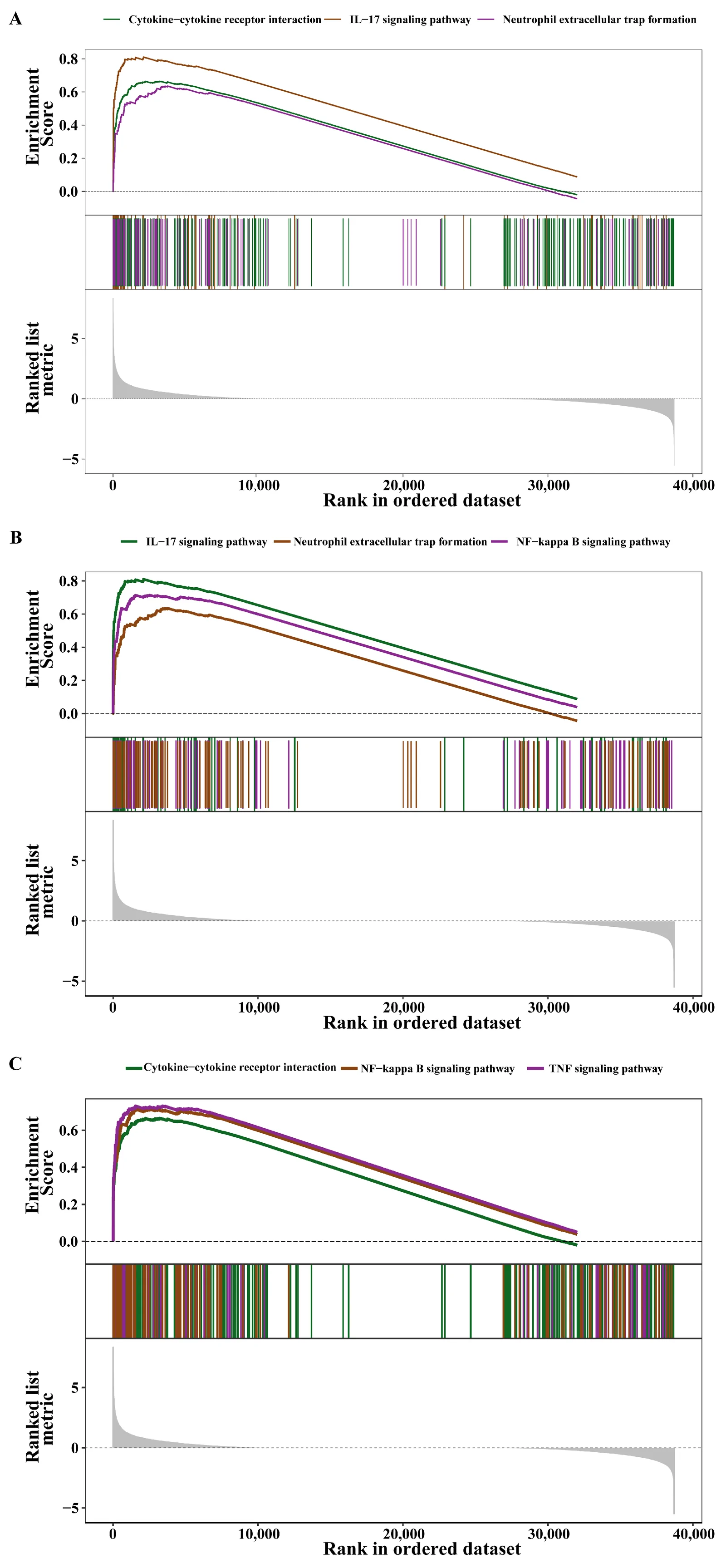
KEGG pathway enrichment analysis of genes co-expressed with hub metabolic genes in SI-ALI. (**A**–**C**) Bar plots of the top enriched KEGG pathways for genes co-expressed with Pmvk (**A**), Slc2a1 (**B**), and Slc7a11 (**C**), respectively.

GSEA was conducted to identify shared inflammatory pathways co-expressed with each metabolic hub gene. Despite distinct core metabolic functions, all three genes converged on overlapping immune-inflammatory signaling programs in SI-ALI. Genes co-expressed with Pmvk were significantly enriched in cytokine-cytokine receptor interaction, IL-17 signaling and neutrophil extracellular trap (NET) formation (Figure 7A). Co-expressed transcripts of Slc2a1 were enriched in IL-17 signaling, NET formation and NF-κB pathways (Figure 7B). Genes correlated with Slc7a11 showed top enrichment in cytokine–receptor crosstalk, NF-κB and TNF signaling cascades (Figure 7C). Consistently overlapping enrichment profiles suggest the shared pathological relevance of the Pmvk-Slc2a1-Slc7a11 triad in association with septic pulmonary inflammation.

**Figure 8:**
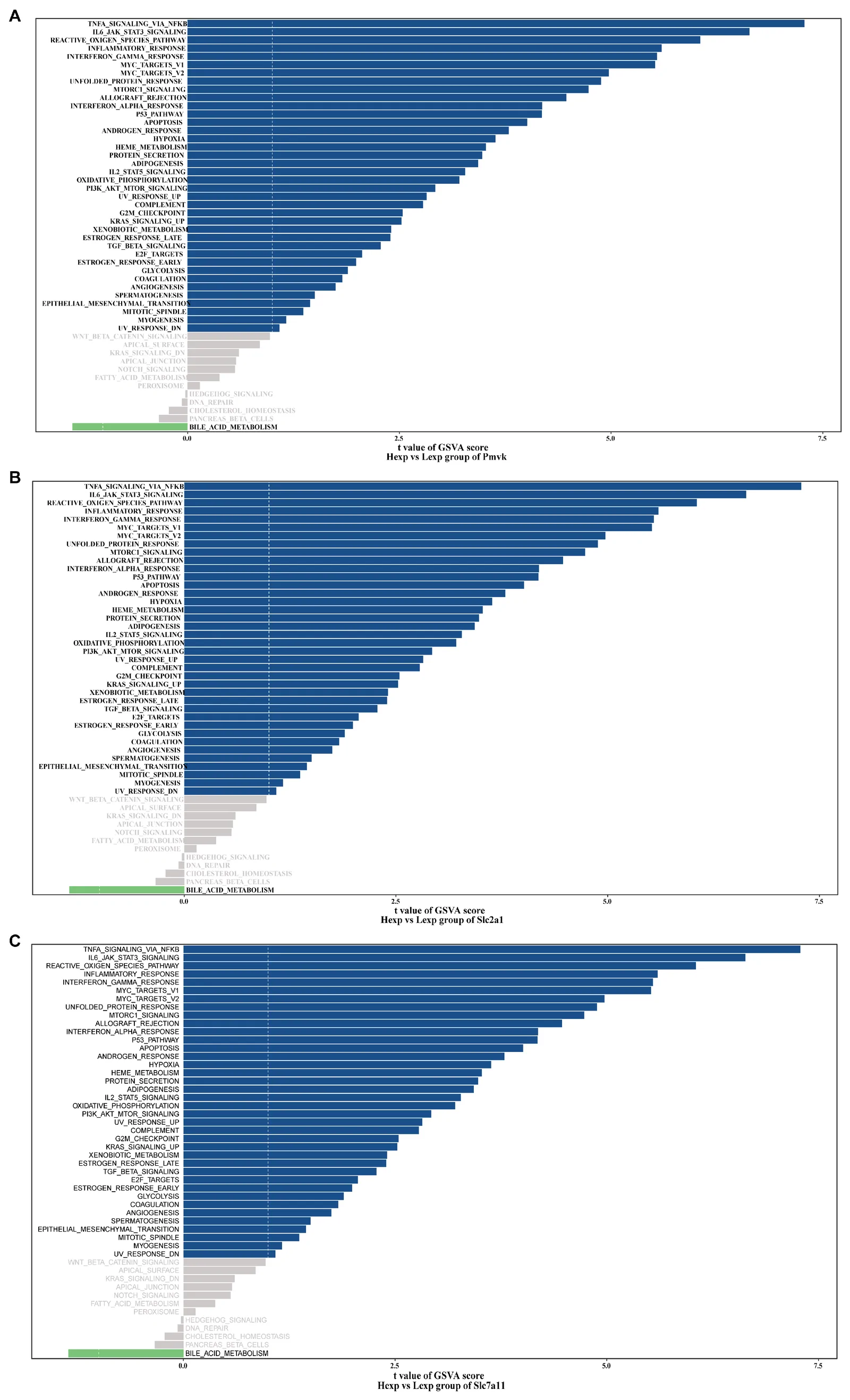
GSVA of hallmark gene sets associated with hub metabolic gene expression. (**A**–**C**) GSVA enrichment scores of hallmark gene sets correlated with Pmvk (**A**), Slc2a1 (**B**), and Slc7a11 (**C**) expression across samples.

GSVA further supported these results. Expression of all three hub genes positively correlated with enrichment scores of hallmark inflammatory gene sets including TNFA_SIGNALING_VIA_NFKB, IL6_JAK_STAT3_SIGNALING and reactive oxygen species (ROS) pathways, suggesting that these metabolic regulators may facilitate neutrophil activation and pro-inflammatory cytokine secretion during SI-ALI progression (Figure 8A–C).

### 3.9. Upstream Regulatory Network Analysis

**Figure 9:**
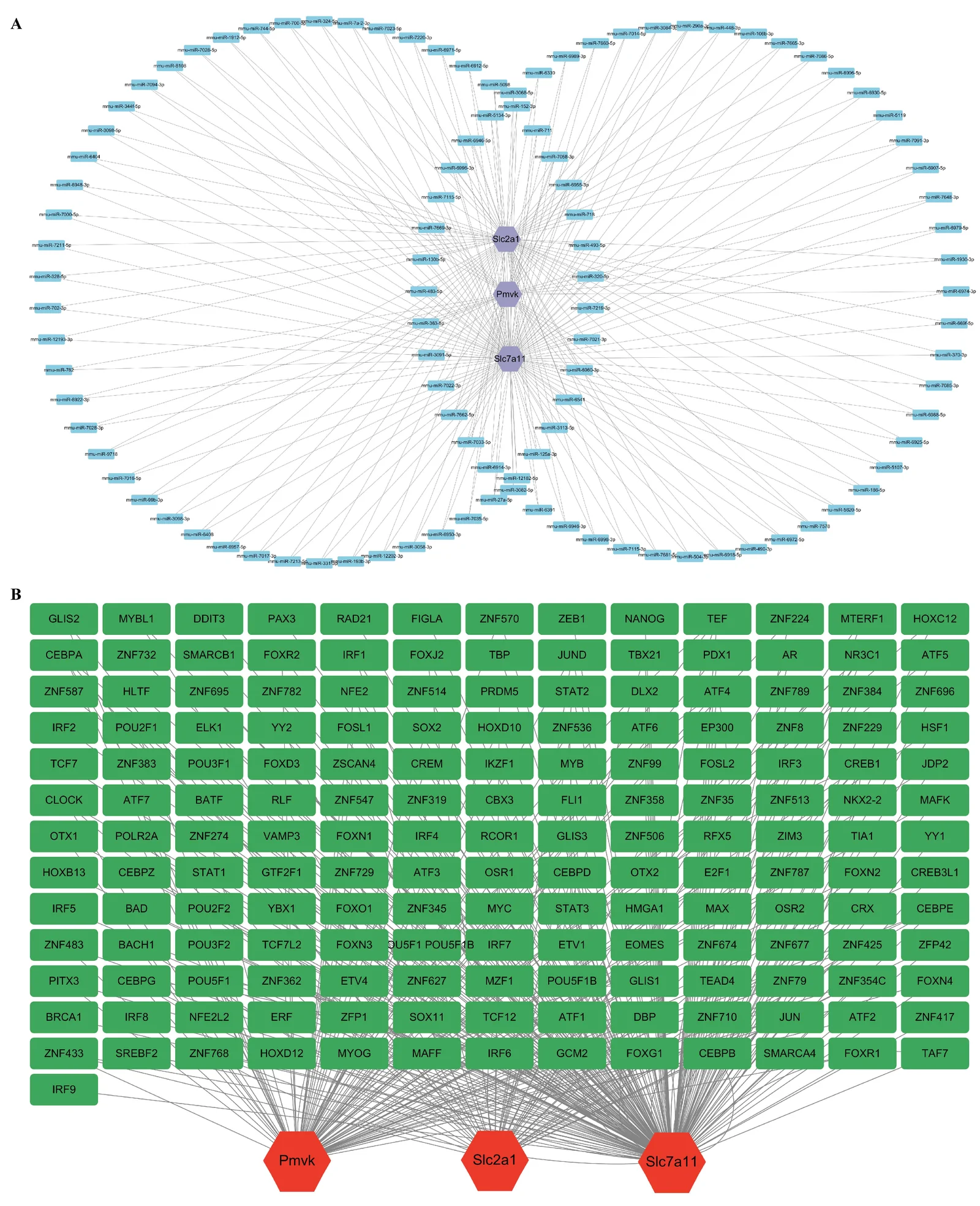
Regulatory network analysis of hub metabolic genes at post-transcriptional and transcriptional levels. (**A**) miRNA–mRNA interaction network constructed from miRWalk predictions, comprising Pmvk, Slc2a1, and Slc7a11 and 106 shared miRNAs predicted to target at least two signature genes. (**B**) Transcription factor–target interaction network identifying 163 putative TFs regulating one or more hub genes, the majority of which co-target multiple members of the triad.

We constructed two layers of upstream regulatory networks to systematically dissect transcriptional and post-transcriptional control of the three metabolic hub genes. At the post-transcriptional level, a miRNA–mRNA interaction network was built based on miRWalk target prediction, comprising 106 shared miRNAs predicted to target at least two of the three hub transcripts (Figure 9A). At the transcriptional layer, we identified 163 transcription factors (TFs) predicted to regulate one or more hub genes; most TFs targeted two or all three members of the triad (Figure 9B). These TFs cover diverse functional categories: canonical inflammatory mediators (Stat3, IRF family, Jun, Myc, E2f1), stress response factors (Ddit3/Chop, Atf3/4/6, Hsf1), developmental and homeostatic TFs (Fox, Cebpa/b/d/g), and chromatin remodelers (Smarcb1, Smarca4, Ep300). Convergent transcriptional and post-transcriptional co-regulation suggests that Pmvk, Slc2a1 and Slc7a11 form a unified stress-induced functional module, consistent with the shared inflammatory pathways identified via GSVA.

### 3.10. Single-Cell Expression Characterization of the Metabolic Signature Genes

**Figure 10:**
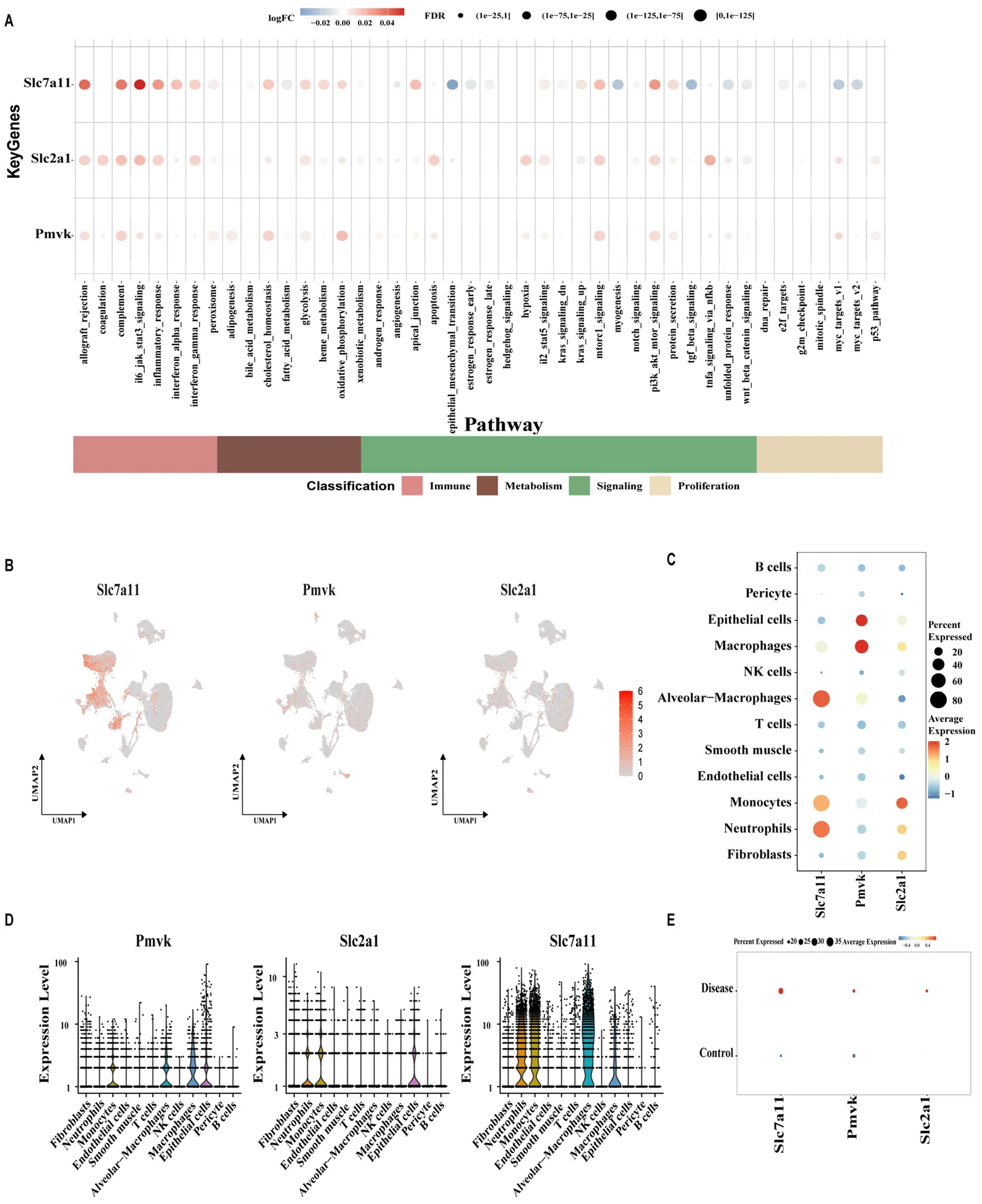
Single-cell expression profiling and pathway association analysis of hub metabolic genes. (**A**) MEBOCOST-based pathway association heatmap showing divergent correlation profiles: Slc7a11 with the strongest positive associations with immune-inflammatory pathways (IL-6/JAK/STAT3, inflammatory response, complement, and interferon α/γ), Slc2a1 with moderate correlations, and Pmvk with limited significant signals. (**B**) UMAP projection of Slc7a11, Slc2a1, and Pmvk expression, revealing myeloid-restricted high expression of Slc7a11 and diffuse distribution of Slc2a1 and Pmvk. (**C**) Dot plot quantifying expression levels and positive cell proportions across annotated cell populations. (**D**) Violin plots confirming single-cell expression distributions and high intra-population heterogeneity of Slc7a11 in myeloid cells. (**E**) Comparison of hub gene expression between control and SI-ALI groups, demonstrating consistent upregulation in the disease group.

We combined MEBOCOST pathway correlation and single-cell expression profiling to characterize cell-type-specific patterns of the three hub genes. MEBOCOST pathway association analysis revealed divergent functional signatures: Slc7a11 exhibited strong positive correlations with multiple immune-inflammatory programs including IL-6/JAK/STAT3 signaling, inflammatory response, complement and interferon pathways. Slc2a1 showed moderate positive associations with immune signaling, while Pmvk displayed only weak, non-significant pathway correlations (Figure 10A).

UMAP single-cell expression visualization demonstrated cell-type-specific expression bias (Figure 10B). Slc7a11 was predominantly restricted to myeloid cells (neutrophils, monocytes, alveolar macrophages) with high transcript abundance, whereas Pmvk and Slc2a1 were broadly distributed across all lung cell types without dominant high-expression populations. Dot plot quantification validated this pattern: Slc7a11 had the highest positive cell proportion and average expression within myeloid subsets; Pmvk was enriched in macrophages and epithelial cells, and Slc2a1 peaked in monocytes (Figure 10C). Violin plots further revealed prominent expression heterogeneity of Slc7a11 within myeloid populations at single-cell resolution (Figure 10D). Consistent with bulk validation, all three hub genes showed significantly elevated expression in SI-ALI relative to control single-cell profiles (Figure 10E). The myeloid-biased expression pattern of Slc7a11 aligns with the widespread pro-inflammatory regulatory roles predicted by pathway enrichment analysis.

### 3.11. Validation of the SI-ALI Mouse Model and qRT-PCR Verification of Inflammatory Mediators and Hub Genes

H&E staining and gross morphological examination were performed to confirm successful establishment of the SI-ALI model. Macroscopically, lung tissues from the sham group presented a normal pale-pink appearance with smooth pleural surfaces and intact lobular structure. In contrast, the lungs of SI-ALI mice were dark red and swollen, exhibiting diffuse congestion and scattered hemorrhagic foci indicative of severe pulmonary edema and parenchymal damage.

**Figure 11:**
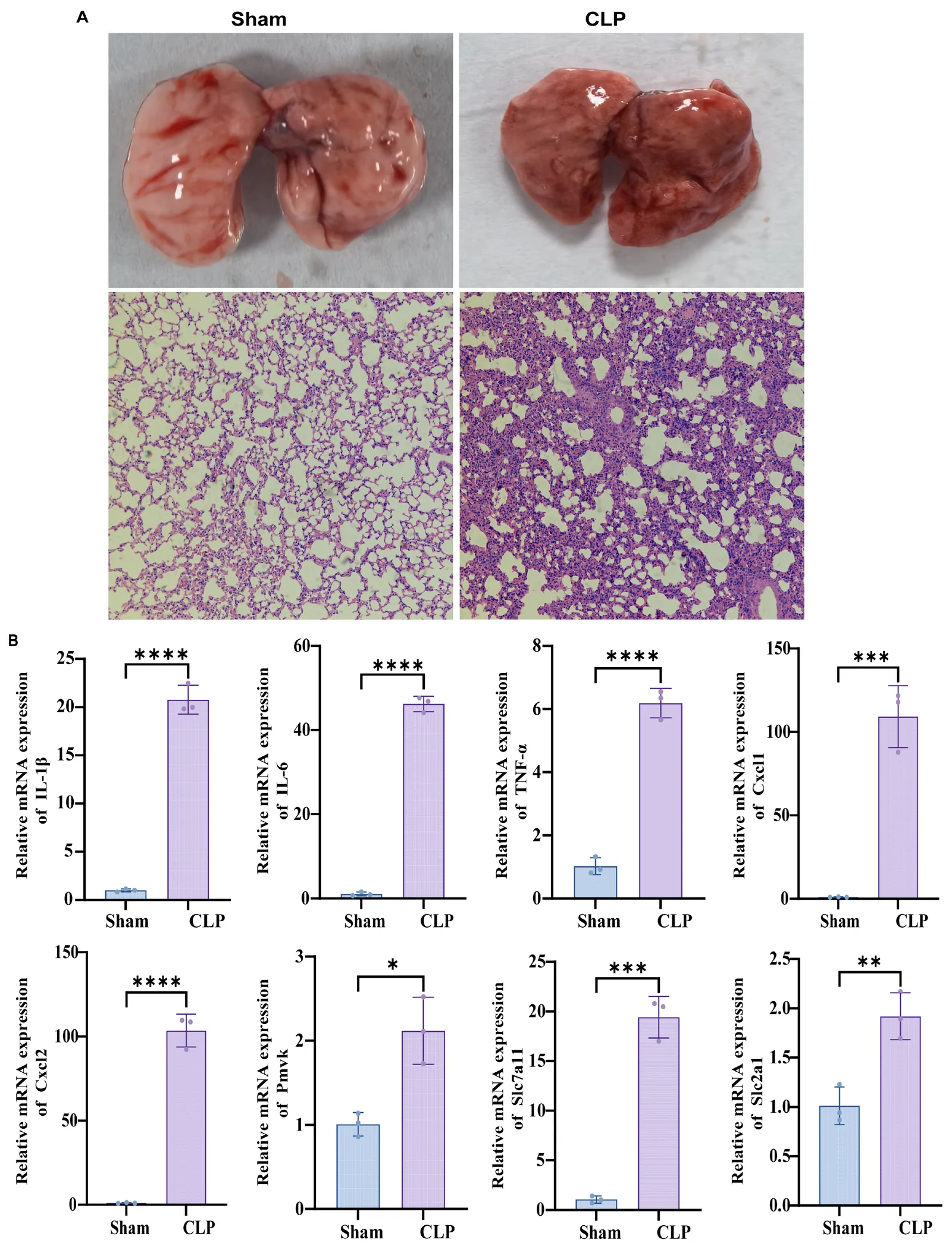
Histopathological and molecular validation of the SI-ALI model (**A**) Gross appearance and H&E staining of lung tissues from sham and SI-ALI mice, showing impaired alveolar architecture and severe inflammatory infiltration in the SI-ALI group. (**B**) qRT-PCR analysis of inflammatory mediators and hub gene mRNA levels in lung tissues (n = 3 per group). Expression was normalized to Gapdh via the 2^−ΔΔCt^ method. Data are mean ± SD; * *p* < 0.05, ** *p* < 0.01, *** *p* < 0.001, **** *p* < 0.0001 vs. sham.

Histologically, the Sham group retained normal pulmonary architecture, characterized by clear alveolar cavities, thin and uniform septa, and only a small number of resident interstitial cells. In contrast, SI-ALI lungs exhibited hallmark features of acute lung injury, including markedly thickened alveolar septa due to tissue edema and extensive inflammatory cell infiltration, abundant inflammatory cells accumulating within alveolar cavities and the interstitium, and focal disruption of alveolar structures (Figure 11A). These findings verified the successful establishment of the SI-ALI model and were consistent with the immune infiltration characteristics revealed by transcriptomic analysis. qRT-PCR was subsequently performed to validate the mRNA expression levels of pro-inflammatory mediators and three hub genes (Slc7a11, Slc2a1, and Pmvk) in lung tissues of Sham and SI-ALI mice (n = 3 per group). As shown in Figure 11B, compared with the Sham group, the mRNA levels of canonical pro-inflammatory cytokines (Il1β, Il6, and Tnfα) were robustly upregulated in the model group. Meanwhile, the neutrophil chemoattractants Cxcl1 and Cxcl2 were also markedly elevated in SI-ALI lung tissues. Notably, all three screened hub genes were significantly overexpressed in the SI-ALI group, among which Slc7a11 showed the most prominent upregulation. These results were consistent with the transcriptomic profiling data, further verifying the reliability of the identified hub genes and indicating their potential roles in mediating sepsis-induced metabolic reprogramming and acute lung injury progression.

### 3.12. Virtual Knockout Simulation of Hub Genes

In silico virtual knockout simulation was performed using scTenifoldKnk to predict transcriptomic changes following depletion of each hub gene.

**Figure 12:**
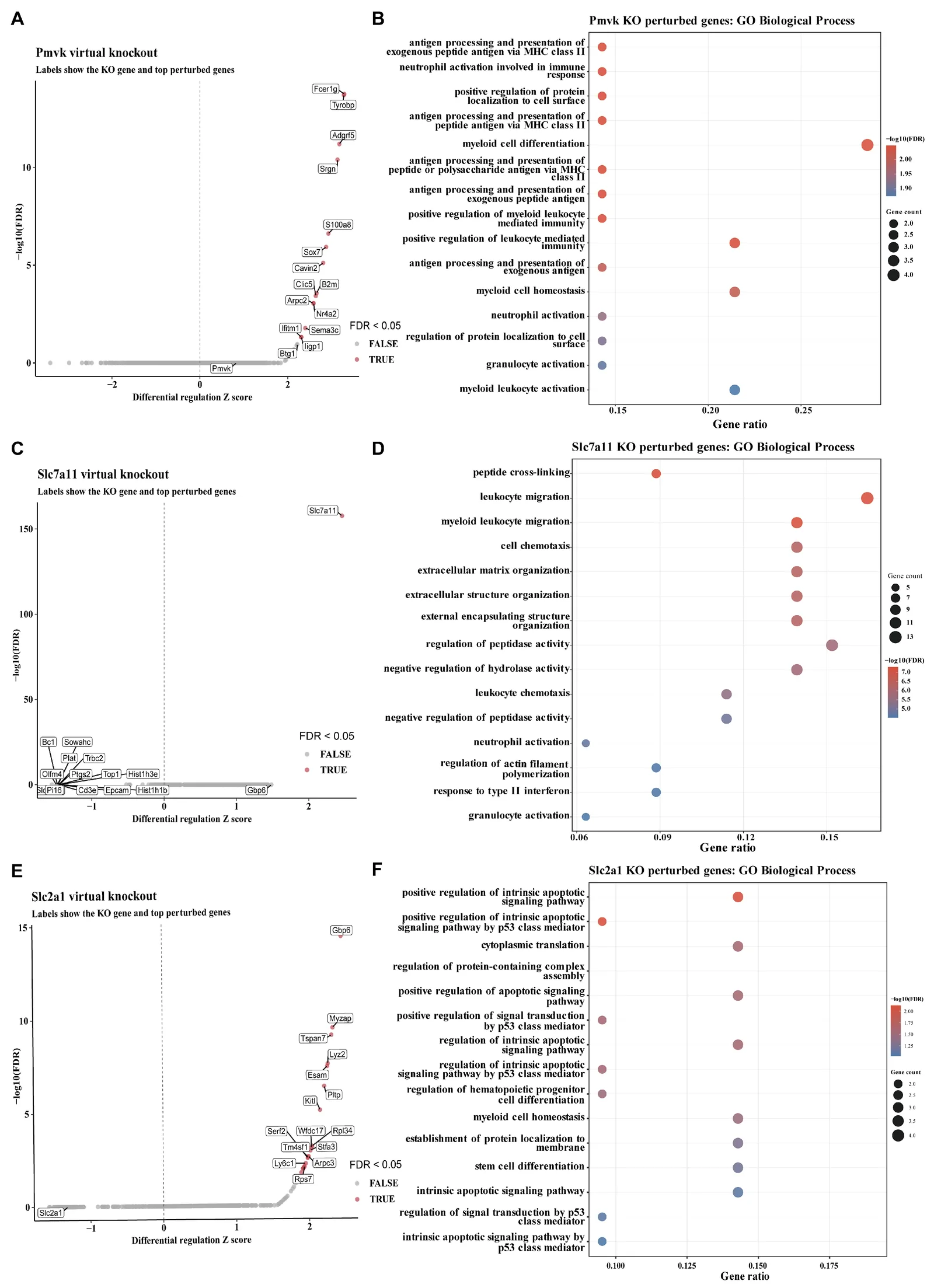
scTenifoldKnk virtual knockout results for three hub genes. (**A**,**C**,**E**) Perturbed gene distribution plots for Pmvk, Slc7a11, Slc2a1. (**B**,**D**,**F**) GO BP enrichment dot plots of each knockout group.

Virtual knockout of Pmvk identified 14 significantly perturbed genes (Figure 12A). These genes were enriched across 519 GO Biological Process (BP) terms, with top enriched pathways covering MHC class II-mediated antigen presentation, neutrophil and myeloid cell activation. This pattern implies Pmvk may be implicated in crosstalk between adaptive and innate immune compartments (Figure 12B). The top perturbed genes included Fcer1g, Tyrobp, Itgax, Itgam, Ckb, and Sema4d.

Virtual knockout of Slc2a1 yielded 22 significantly perturbed genes (Figure 12E), including representative transcripts such as Lyz2, Gpx1, Selp, Mcm6, Tspan7, Adam10, and Alox5. These perturbed genes were associated with 218 enriched GO Biological Process terms, with top enriched pathways focusing on intrinsic apoptotic signaling, p53-mediated stress response, and cytoplasmic translation (Figure 12F).

Virtual knockout of Slc7a11 produced the most extensive transcriptomic perturbations, with 80 significantly altered genes and 694 enriched GO BP terms (Figure 12C). The dominant enriched processes were leukocyte migration, myeloid chemotaxis, ECM remodeling, and granulocyte/neutrophil activation. These observations imply Slc7a11 may modulate immune cell trafficking and ECM homeostasis (Figure 12D). Top perturbed genes included Mpo, Lyz2, S100a8, S100a9, Mmp9, Mmp2, Tnf, Vcam1, Il1r1, and Col3a1.

Cross-comparison of the three hub gene knockout simulations indicated that Slc7a11 ablation induced more extensive transcriptional alterations, consistent with its high centrality within the MEBOCOST-predicted alveolar macrophage–neutrophil metabolic communication network. The perturbation of leukocyte migration pathways after Slc7a11 knockout suggests a pronounced effect on this signaling axis, consistent with its predicted role in AM-derived metabolic signaling toward neutrophils.

Collectively, these computational perturbation analyses provide predictive functional evidence linking the three hub genes to immune activation, metabolic stress response and inflammatory signaling in SI-ALI. All regulatory conclusions drawn from virtual knockout are purely in silico predictions and require independent wet-lab gene knockout experiments for definitive validation.

## 4. Discussion

SI-ALI is characterized by concurrent immune dysregulation and profound metabolic reprogramming, yet how metabolite-mediated signaling coordinates these two processes at the intercellular level remains incompletely understood. By integrating scRNA-seq with protein- and metabolite-level intercellular communication analyses, the present study reconstructed a metabolic-immune crosstalk network in SI-ALI in which alveolar macrophages (AMs) and neutrophils form the central axis.

The central role of AMs as metabolic hubs in septic lung injury is rooted in their unique anatomical position and long-lived tissue-resident programming [23,24,25]. Stationed at the air–tissue interface, AMs are the first immune sentinels to encounter pathogens, inhaled toxins, and alveolar damage signals [23]. Unlike short-lived infiltrating monocytes and neutrophils that are optimized for acute bactericidal function, AMs are adapted for continuous environmental sampling, signal relay, and metabolic conversion [23,24]. This sentinel function requires a broad repertoire of metabolic enzymes and membrane transporters [23,24], enabling AMs to import, process, and export diverse metabolites. We therefore propose that AMs do not merely respond to inflammatory cues but may actively convert stromal- and pathogen-derived metabolic inputs into neutrophil-directed signaling outputs. This observation implies that AMs may act as metabolic integrators that potentially link protein- and metabolite-mediated communication layers, with the CXCL–CXCR2 chemotactic axis and AM-derived metabolite export constituting two complementary arms of this hierarchical control. Histopathological examination confirmed the classic SI-ALI phenotypes of alveolar septal thickening and massive myeloid cell infiltration, which is in line with the AM-driven myeloid recruitment and immune remodeling observed at the transcriptomic level.

Two metabolite–sensor axes are proposed to explain how AM-derived metabolic outputs may directly amplify neutrophil inflammatory function. The iron–Slc40a1 axis is predicted to function as a ferroptosis-to-ROS relay. AMs express ferroportin (Slc40a1), the sole cellular iron exporter, which releases ferrous iron into the alveolar space [26]. Neutrophils take up this iron via Slc11a2 (DMT1), where it drives Fenton-type reactions and NADPH oxidase-dependent ROS production [27,28]. Rather than merely damaging AMs themselves, exported iron may thus be repurposed as a metabolic messenger that potentially propagates neutrophil cytotoxicity and oxidative tissue injury. In parallel, the LTB4–Ltb4r1 axis is predicted to constitute a lipid mediator circuit that may facilitate neutrophil recruitment and activation. LTB4, a 5-lipoxygenase-derived eicosanoid [29] synthesized by AMs [30], signals through LTB4R1 on neutrophils to reinforce CXCL-driven chemotaxis [29,31], promote degranulation [32], and induce NET formation [33]. Together, iron is predicted to fuel the ROS engine and LTB4 to provide directional guidance, potentially creating a dual-metabolite inflammatory circuit in which AMs may lower the activation threshold and steer neutrophil effector function.

RF and LASSO independently converged on Pmvk, Slc2a1, and Slc7a11, identifying these transporters and enzymes as nodal points within the SI-ALI metabolic network. Each is implicated in a distinct arm of myeloid activation: Pmvk channels mevalonate flux toward protein prenylation, a post-translational modification required for Rho GTPase membrane targeting [34] and NLRP3 inflammasome assembly [14]; Slc2a1 (GLUT1) drives the glycolytic shift that sustains rapid ATP generation and succinate accumulation, coupling metabolic flux to inflammatory signaling [9,35]; and Slc7a11 mediates cystine–glutamate exchange, linking antioxidant defense to paracrine glutamate signaling [36]. Because these genes were identified through MEBOCOST-inferred metabolic crosstalk and validated in an independent cohort, their upregulation likely reflects active participation in intercellular communication rather than merely cell-autonomous adaptation. Gong et al. recently demonstrated that macrophages function as dominant regulators of the lung cellular network during sepsis through ZBP1-mediated metabolic reprogramming and NLRP3 inflammasome activation [37]; our findings extend this concept by identifying candidate metabolic conduits—Pmvk, Slc2a1, and Slc7a11—through which AMs may exert hierarchical regulation over neutrophil effector functions. The transcriptional upregulation of this three-gene panel was further corroborated by in vivo qRT-PCR validation, reinforcing the robustness of our bioinformatic screening.

Transcriptionally, Slc7a11 appears to be the most pleiotropic of the three hub genes and is inferred to be associated with three pathological dimensions of SI-ALI. First, by importing cystine—the rate-limiting substrate for glutathione (GSH) biosynthesis—Slc7a11 upregulation may modulate intracellular redox homeostasis and susceptibility to lipid-peroxidation-driven ferroptosis [36]. Second, the reverse transport of glutamate is predicted to generate a paracrine inflammatory signal: extracellular glutamate may activate NMDA receptors and mGluRs on pulmonary immune and stromal cells, amplifying local cytokine and chemokine cascades [38,39]. Third, Slc7a11 is co-expressed with both pro-inflammatory mediators (Il6 and Cd28) and immunosuppressive checkpoints (Il10, Tigit, Tgfbr1), a signature that approximates MDSC-like polarization in sepsis [40]. This tripartite role may provide a mechanistic explanation for the hallmark immune paradox of concurrent hyperinflammation and systemic immunoparalysis. From a translational perspective, Slc7a11/system xc^−^ is a candidate modulatory target, but its net effect is highly context-dependent [40]. Slc7a11 may influence the inflammation–ferroptosis balance by regulating cystine uptake, GSH synthesis, redox homeostasis, and glutamate release; consequently, the therapeutic direction—whether to inhibit or enhance its activity—cannot be inferred from expression data alone and must be validated in a cell-type- and stage-specific manner.

The three hub genes do not operate in isolation. Despite weak single-gene pathway correlations, GSEA and GSVA indicate that Pmvk, Slc2a1, and Slc7a11 converge on shared inflammatory pathways—IL-17 signaling, NF-κB/TNF-α signaling, and NET formation—suggesting they form a co-regulated metabolic–immune module. This convergence is underpinned by extensive shared transcriptional and post-transcriptional regulation, including miRNAs and transcription factors linking inflammatory, stress, and chromatin-remodeling programs. Such coordinated control explains how genes with distinct metabolic roles can be synchronously activated by convergent danger signals, creating a functionally integrated module that aligns metabolic flux with immune effector output.

To functionally interrogate this module in silico, we performed scTenifoldKnk-mediated virtual knockout for each hub gene. Pmvk ablation predominantly disrupted antigen presentation and myeloid cell adhesion, whereas Slc2a1 deficiency enriched p53-mediated apoptotic pathways—a finding consistent with glucose starvation-induced oxidative stress. Notably, Slc7a11 depletion elicited the most extensive transcriptional perturbations, broadly affecting leukocyte migration, extracellular matrix (ECM) remodeling, and granulocyte activation. The observed perturbation magnitude (Slc7a11 > Slc2a1 > Pmvk) exhibited a striking correspondence with the topological hierarchy of the metabolic crosstalk network, computationally substantiating that nodal centrality determines functional reach. We emphasize that these inferences remain purely predictive and necessitate rigorous wet-lab validation.

This coordinated signature may therefore help stratify the metabolic–immune status of patients with sepsis.

Several limitations of the present study should be acknowledged. First, all metabolic communication outputs are computationally inferred from transcriptomic profiles using MEBOCOST, and direct metabolite quantification (e.g., LC-MS/MS measurement of labile iron, LTB4, and cystine/glutamate and GSH/GSSG ratios in BALF and lung tissue) was not performed. Second, protein-level validation and cell-specific co-localization of the hub genes—including multiplex immunofluorescence co-staining with F4/80 (AMs) and Ly6G (neutrophils) and Western blot quantification in isolated AMs—were not conducted. Third, while virtual knockout simulation using scTenifoldKnk provided in silico functional evidence supporting the regulatory roles of the hub genes in immune cell activation and leukocyte migration, direct wet-lab loss-of-function studies (e.g., AM-specific knockdown of Slc7a11, Slc2a1, or Slc40a1/Ltb4r1) were not performed. Finally, the clinical translational value of the metabolic signature warrants evaluation in larger independent SI-ALI cohorts. Collectively, these limitations highlight the directions for future validation, ranging from targeted metabolite quantification and protein-level validation to loss-of-function experiments and assessment in larger independent SI-ALI cohorts.

## 5. Conclusions

In summary, our findings reconstruct a metabolite-mediated communication cascade flowing sequentially from stromal cells to AMs and onward to neutrophils, bridging metabolic reprogramming and immune dysregulation in the context of SI-ALI. AMs serve as metabolic hubs: their anatomical position and broad transporter/enzyme repertoire enable them to integrate stromal-derived cues and convert them into neutrophil-directed signaling outputs. The iron–Slc40a1 and LTB4–Ltb4r1 axes are proposed to define the two principal routes by which AM-derived metabolites may amplify neutrophil inflammation, with iron predicted to fuel ROS generation and LTB4 driving chemotaxis and activation. Among the three hub genes, Slc7a11 appears as the most translationally relevant, simultaneously bridging redox homeostasis, inflammatory amplification, and immunosuppressive feedback—the three pillars of the SI-ALI immune paradox. While the computational predictions are supported by independent published experimental evidence and in silico virtual knockout simulation, these findings require wet-lab functional validation through targeted metabolomics, protein-level verification, and loss-of-function studies. Modulating the Slc7a11-mediated metabolic rheostat along the AM–neutrophil axis may therefore offer novel therapeutic strategies, but its precise direction must be validated in a cell-type- and stage-specific manner.

## Data Availability

The original gene expression data analyzed in this work are openly available in the Gene Expression Omnibus (GEO) database at https://www.ncbi.nlm.nih.gov/geo/ (accessed on 25 March 2026), and can be retrieved using the accession numbers GSE207651 and GSE269740.

## Supplementary Materials

The following supporting information can be downloaded at: https://www.mdpi.com/article/10.3390/genes17091019/s1, Figure S1: Quality control, preprocessing, and batch correction of scRNA-seq data.

## Appendix A

**Table A1:** Primer sequences for RT-qPCR target genes and inflammatory cytokines.

Gene	Forward Primers (5′–3′)	Reverse Primers (5′–3′)
Pmvk	CTTGGAGGTAACATCTGTGCTC	GTGCTCGCATCCAGAAGTCTC
Slc2a1	CAGTTCGGCTATAACACTGGTG	GCCCCCGACAGAGAAGATG
Slc7a11	GGCACCGTCATCGGATCAG	CTCCACAGGCAGACCAGAAAA
Cxcl1	CTGGGATTCACCTCAAGAACATC	CAGGGTCAAGGCAAGCCTC
Cxcl2	CCAACCACCAGGCTACAGG	GCGTCACACTCAAGCTCTG
TNF-α	GGTGCCTATGTCTCAGCCTCTT	GCCATAGAACTGATGAGAGGGAG
IL-6	TAGTCCTTCCTACCCCAATTTCC	TTGGTCCTTAGCCACTCCTTC
IL-1β	GCAACTGTTCCTGAACTCAACT	ATCTTTTGGGGTCCGTCAACT
GAPDH	AGGTCGGTGTGAACGGATTTG	TGTAGACCATGTAGTTGAGGTCA
