# Supplementary material for: Single-Cell RNA Sequencing Combined with MEBOCOST Reveals Alveolar Macrophage-Mediated Immunometabolic Communication Mechanisms in Sepsis-Induced Acute Lung Injury
Source: Genes (Basel). 2026 Aug 27;17(9):1019. doi: 10.3390/genes17091019 (PMC13606234; doi:10.3390/genes17091019)
Supplement: Supplementary file 1 [file genes-17-01019-s001.zip › genes-4449729-supplementary.pdf]

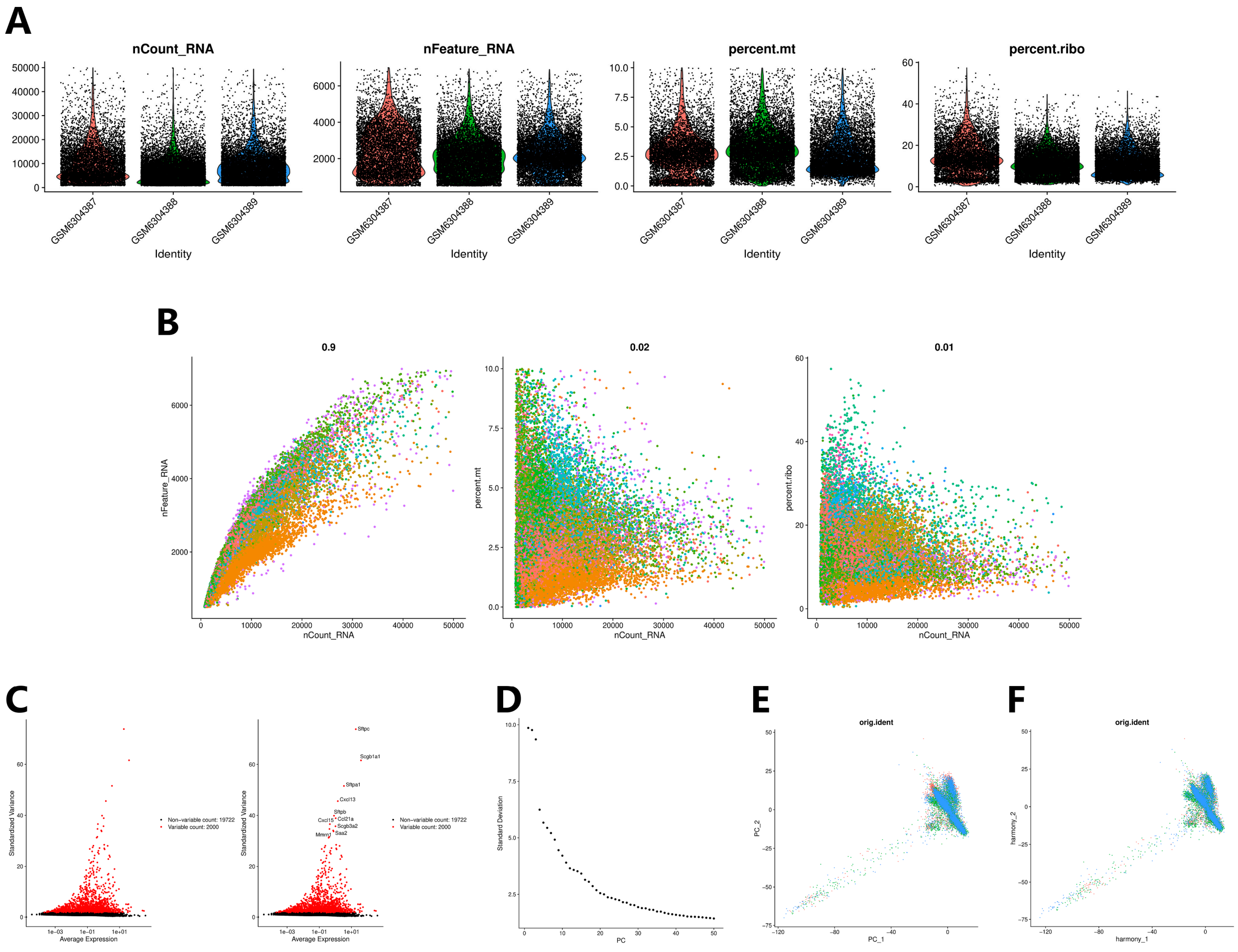

Figure S1. Quality control, preprocessing, and batch correction of scRNA-seq data
(A) Violin plots showing the distribution of nCount_RNA, nFeature_RNA, percent.mt, and percent.ribo across three samples, demonstrating consistent inter-sample quality. (B) Correlation analysis of QC metrics; nFeature_RNA and nCount_RNA were strongly correlated (R = 0.90), whereas percent.mt and percent.ribo showed negligible correlation with nCount_RNA (R = 0.02 and R = 0.01, respectively).(C) Top highly variable genes (HVGs) identified after normalization, highlighting pulmonary epithelial markers (Sftpc, Scgb1a1, Sftpa1, Sftpb, Scgb3a2) and the inflammatory chemokine Cxcl13. (D) Elbow plot indicating that the first 20 principal components captured the majority of biological variance. (E) PCA plot prior to batch correction, showing partial sample-specific clustering. (F)PCA plot following Harmony integration, demonstrating effective removal of batch effects.
